# Metabolization of human milk oligosaccharides by *Streptococcus pneumoniae*

**DOI:** 10.1038/s41467-026-77970-7

**Published:** 2026-09-30

**Authors:** Katharina Bacher, Ustina Martysiuk, Greicy K. Bonifacio-Pereira, Johann Mignolet, Vincent de Bakker, Melika Ghazaghi, Lukas Troxler, Tabea N. Bommer, Ilche Gjuroski, Julien Furrer, Loretta Müller, Judit Burgaya, Nadja Mostacci, Daniel R. Neill, Jan-Willem Veening, Markus Hilty

**Affiliations:** 1 Institute for Infectious Diseases, Faculty of Medicine, University of Bern, Bern, Switzerland; 2 Graduate School of Cellular and Biomedical Science, University of Bern, Bern, Switzerland; 3 Division of Molecular Microbiology, School of Life Sciences, University of Dundee, Dundee, UK; 4 Department of Fundamental Microbiology, Faculty of Biology and Medicine, University of Lausanne, Lausanne, Switzerland; 5 Department of Microbiology, Harvard Medical School, Boston, MA USA; 6 Department of Chemistry, Biochemistry and Pharmacy, University of Bern, Bern, Switzerland; 7 Division of Pediatric Respiratory Medicine and Allergology, Department of Pediatrics, Inselspital, Bern University Hospital, Bern, Switzerland; 8 Lung Precision Medicine (LPM), Department for BioMedical Research (DBMR), University of Bern, Bern, Switzerland

**Keywords:** Bacterial adhesion, Bacterial genes, Pathogens, Glycobiology, Metabolic pathways

## Abstract

It is unclear whether human milk oligosaccharides (HMOs) can be used as a nutrient source by *Streptococcus pneumoniae*. Here, we investigate whether and how *S. pneumoniae* metabolizes HMOs and whether these compounds inhibit pneumococcal adherence. We find that *S. pneumoniae* can metabolize several HMOs, except for 2’-fucosyllactose (2’FL). The process is regulated by the carbon catabolite repression system. Transcriptome analyses showed reduced expression of the *lac* operon I (*lacABCD*) in bacteria grown with two tested HMOs when compared to lactose (Lac). Genes for the Leloir pathway and exoglycosidases were upregulated. A genome-wide CRISPR interference screen showed that the *lac* operons I and II (*lacEFG-2*) are essential for metabolizing HMOs. Lac and non-metabolizable 2’FL were found to inhibit pneumococcal adherence to host cells in vitro. Importantly, 2’FL showed a stronger inhibitory effect compared to Lac in vivo. Further research is warranted to investigate whether the non-metabolizable 2’FL can be used to inhibit pneumococcal adherence.

## Abstract (web summary)

It is unclear whether human milk oligosaccharides (HMOs) can be used as a nutrient source by *Streptococcus pneumoniae*. Here, Bacher et al. report that these bacteria can metabolize several HMOs, identify genes and pathways involved in the process, and show that a non-metabolizable HMO inhibits pneumococcal adherence to host cells.

## Introduction

*Streptococcus pneumoniae* is part of the upper airway microbiota, with the nasopharynx/oropharynx of 40–95% of infants and 10–25% of adults being colonised at some point in their lives^1–3^. Colonisation can lead to invasive pneumococcal diseases, particularly in young children (< 5 years old) or older adults (> 65 years old)^4^. *S. pneumoniae* encounters a vast variety of carbohydrates in the various niches it can occupy on its human host. Monosaccharides are imported via a set of different transporters, mainly belonging to either the ATP-binding cassette (ABC) transporter superfamily or the phosphoenolpyruvate phosphotransferase system (PTS)^5^. *S. pneumoniae* relies exclusively on carbohydrates as a carbon source (C-source) and devotes 30% of all its transport mechanisms to their import^6,7^. After being imported, monosaccharides are metabolized by the Embden–Meyerhof–Parnas pathway or the pentose phosphate pathway, both of which are involved in energy generation, or they are converted to UDP-glucose and UDP-galactose, which serve as precursor molecules for the biosynthesis of the polysaccharide capsule^8^. *S. pneumoniae* possesses a carbon catabolite repression (CCR) system, allowing it to use its preferred C-sources by repressing the transcription of genes whose products are involved in the use of other, less preferred carbohydrates^9–13^. Importantly, pneumococci can metabolize a vast range of host-derived and dietary glycans^5,14,15^. This versatility in C-source utilisation, enabled by exoglycosidases, is a major factor in pneumococcal virulence^16–18^, as the respiratory tract is rich in diverse glycoconjugates. Exoglycosidases have different affinities according to substrates primarily due to the specific and complex structure of their active sites, which are designed to recognize unique glycan (sugar) types and the specific anomeric linkages at the non-reducing ends of carbohydrate chains^19^. In particular, it has been demonstrated that beta-galactosidase (BgaA), neuraminidase (NanA) and beta-N-acetylglucosaminidase (StrH) act sequentially to remove sialic acid (Sia), galactose (Gal), and N-acetylglucosamine (GlcNAc), thereby exposing Mannose on human glycoproteins that binds to the pneumococcus and protects the airways^20^. Mannose binding to the pneumococcus activates the lectin pathway of the complement system, which ultimately leads to phagocytosis, clearing the pathogen from the airway^21^. Therefore, there is a fine balance between cleaving/metabolizing host-derived carbohydrates and using them to ensure efficient adherence to the host^15^.

*S. pneumoniae* has several carbohydrate transporters, but investigating their essentiality by studying loss-of-function mutant strains has so far been challenging due to substrate redundancy^5^. A deeper understanding is desirable because transporters could be important drug targets, as they are specific for prokaryotes^5^. Measuring gene expression at the transcriptomic level is powerful to better understand regulation, but does not allow investigating essentiality. In contrast, CRISPR interference sequencing (CRISPRi-seq) is an unbiased method to measure genome-wide effects on cell fitness^22^. Combining these complementary methods can therefore provide a system-level perspective for comprehensive gene function studies^13^.

It has been hypothesized that *S. pneumoniae* could encounter dietary glycans in the oropharynx during transmission or colonization, or as components of another bacterial capsule^15^. Possibly, there is also an increased availability of dietary glycans, including lactose (Lac) and human milk oligosaccharides (HMOs), in the naso- and oropharynx during breastfeeding, due to the close link to the oral cavity^23^. Importantly, it has also been suggested that HMOs are absorbed into the bloodstream and excreted in urine, as they were detected in the blood and urine of breastfed infants, infants receiving HMO-fortified formula, and adults receiving HMO supplements, demonstrating their systemic availability^24^. Thus, HMOs are likely to also play a role as dietary glycans during invasive pneumococcal disease and might also impact the outcome of the disease. In addition, HMOs might have potential therapeutic utility, as has recently been shown for group B *Streptococcus*^25^. By separating different HMO fractions, the authors found the bacteriostatic activity to be confined to specific non-sialylated HMOs and synergy with a number of conventional antibiotic agents.

Biosynthetically, HMOs follow a blueprint, wherein every oligosaccharide (OS) contains Lac at its reducing end^26^. Human breast milk contains three major HMO types: fucosylated HMOs (35–50%), sialylated HMOs (12–14%), and non-fucosylated neutral HMOs (42–55%)^27^. There are unanswered questions regarding the access to and processing of dietary glycans by *S. pneumoniae*. For instance, strains are carrying genes for fucose utilization but are unable to grow with Fucose as the sole C-source^15^.

Here, we identify which HMOs are metabolized by *S. pneumoniae* and under which growth conditions this occurs. Furthermore, we describe the genes required for HMO metabolization and profile gene expression during growth on HMOs, using CRISPRi-seq and transcriptomic sequencing. Finally, we show that HMOs, and the non-metabolizable 2′-fucosyllactose (2′FL) in particular, act as powerful inhibitors for pneumococcal adherence in vitro and in vivo.

## Results

### In vitro HMO metabolization depends on the culture medium composition

**Fig. 1:**
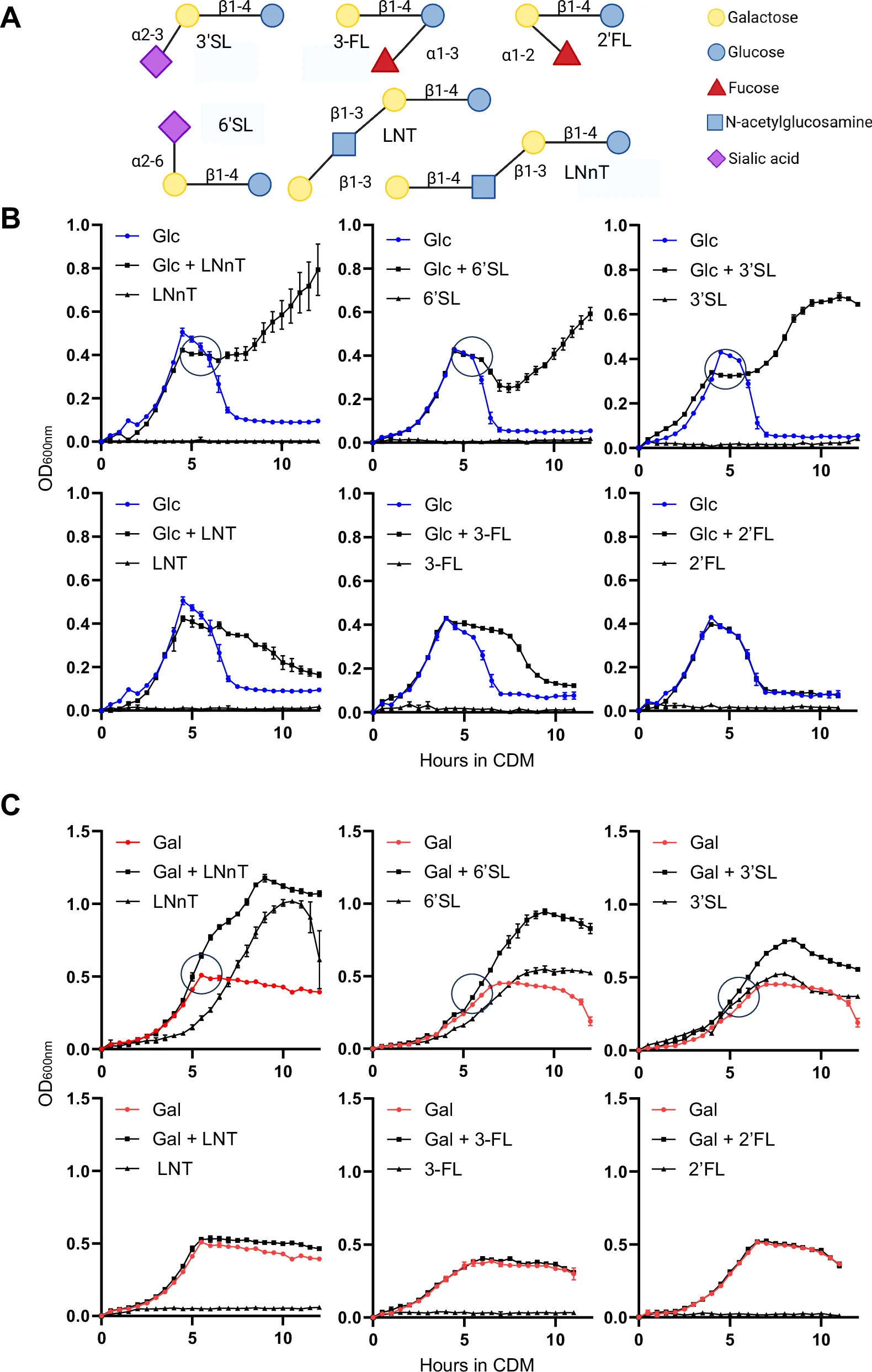
Metabolization of six human milk oligosaccharides (HMOs) by *S. pneumoniae*. Growth was measured after pre-growth in either glucose (Glc) or galactose (Gal) in a Lacks medium, and subsequent growth with single HMOs as the sole carbon source (C-source) or as diauxic growth in chemically defined medium (CDM). In (**A**), the structures of the used HMOs for growth are shown. Created in BioRender. Hilty, M. (2026) https://BioRender.com/xxwrpyd. In (**B**), pre-growth of 106.66 (serotype 6B) in Glc with subsequent growth in different HMOs (2′FL 2′-fucosyllactose, 3-FL 3-fucosyllactose, 3′SL 3′-sialyllactose, 6′SL 6′-sialyllactose, LNnT Lacto-N-neotetraose, and LNT Lacto-N-tetraose), each used as the sole C-source or as diauxic growth, is displayed. The growth of the strain using Glc is also displayed as a reference (blue lines). In (**C**), pre-growth of 106.66 in Gal with subsequent growth in the 6 different HMOs, each used again as the sole C-source or as diauxic growth, is displayed. The growth of the strain using Gal is also displayed as a reference (red lines). The mean of three replicates is plotted ± SEM. The circles illustrate the different fate of growth using either Glc or Gal during diauxic growth.

To dissect the genuine importance of HMOs in *S. pneumoniae* metabolism, we first characterised the growth dynamics of strains 106.66 (serotype 6B), D39 (serotype 2) and its unencapsulated derivative R6 in the presence of six purified HMOs: 2′FL, 3-fucosyllactose (3-FL), Lacto-N-tetraose (LNT), Lacto-N-neotetraose (LNnT), 3′-sialyllactose (3′SL), and 6′-sialyllactose (6′SL) (Table 1, Figs. 1A–C and S1, and Supplementary Data 1).

**Table 1:** *Streptococcus pneumoniae* strains used in this study

ID	Description	Source
Wild-type		
106.66	serotype 6B, MLST: 2244, IFIK	Clinical isolate^66,67^
208.41	serotype 7 F, MLST: 191, IFIK	Clinical isolate^66,67^
D39	serotype 2, MLST:595	Lab strain^68,69^
R6	derived from D39; non-typable, UN MLST:595	Lab strain^7,69^
Transformants		
D39Δ*bgaA*	D39Δ*bgaA*::Em^r^	^20^
D39Δ*nanA*	D39Δ*nanA*::Cm^r^	^70^
D39Δ*strH*	D39Δ*strH::*Cm^r^	
R6Δ*bgaA*	UN, R6Δ*bgaA*::Em^r^	^20^
R6Δ*nanA*	UN, R6Δ*nanA*::Cm^r^	^20^
FP382	UN, background DP1004 - parental strain of R6, SP1185Δ*lacE-2*::Km^r^ (PTS system, Lactose-specific IIBC)	^5^
FP415	UN, background DP1004 - parental strain of R6, spr0279ΔEIIA::Km^r^-*aphIII-rpsL*^+^-Sm^s^ (SP0305, *celB*, beta-glucoside regulator (*rpsL41*) JANUS (EIIA domain))	^5,71^
FP435	UN, background R6, spr0282ΔEIIC::Km^r^ (SP0310, *celD*, PTS EIIC)	^5,72^
VL2212	D39V, serotype 2, *bgaA*::P_tet_-*dCas9sp*, *prsA*::PF6-*tetR*, aTc-inducible	^38^
HL23-1	D39, serotype 2, *bgaA*:: P_tet_-*dCas9sp*, *prsA*::*tetR*, aTc-inducible	This study
HL23-2	R6, UN, *bgaA*:: P_tet_-*dCas9sp*, *prsA*::*tetR*, aTc-inducible	This study
D39Δ*lacG-2*	serotype 2, D39Δ*lacG-2*::Km^r^, 6-phosphate-beta-galactosidase (SP1184, *lacG-2*)	This study
R6Δ*lacG-2*	UN, R6Δ*lacG-2*::Km^r^, 6-phosphate-beta-galactosidase (SP1184, *lacG-2*)	This study
208.41Δ*lacG-2*	serotype 7 F, 208.41Δ*lacG-2*::Km^r^, 6-phosphate-beta-galactosidase (SP1184, *lacG-2*)	This study
208.41_Rev	Serotype 7 F, 208.41 gene-complemented strain	This study

*MLST* multi-locus sequence type. Strains were isolated at the IFIK (Institute for Infectious Diseases, Bern, Switzerland). *Cm*^*r*^ resistance to chloramphenicol, *Em*^*r*^ resistance to erythromycin, *Sm*^*S*^ restored resistance to spectinomycin, *Km*^*r*^ resistance to kanamycin, *UN* unencapsulated, *aTc* Anhydrotetracycline, *P*_*tet*_ represents PT4-lin^73^.

We tested two different pre-growth conditions, including either 5.5 mM glucose (Glc) or Gal, before investigating individual and diauxic growth for each of the HMOs (Fig. 1B, C). In the presence of Glc, subsequent metabolization of LNnT, 6′SL and 3′SL was only observed after cells began to lyse (Fig. 1B).

In contrast, continuous growth was noted in the presence of Gal (Fig. 1C). Consequently, using HMOs as single C-source, metabolization was not observed if pre-grown in Glc, but the opposite was true in Gal for LNnT, 6′SL, and 3′SL. Diauxic growth for LNT + Glc and 3-FL + Glc showed delayed lysis of cells, but generally no growth was observed.

Using higher concentrations of HMOs, we found that only LNT allows growth as the sole C-source if Glc is used in the pre-growth medium (Fig. S1A). In contrast, using Gal for pre-growth, we found that all tested HMOs facilitate growth except 2′FL (Fig. S1B).

However, if the concentrations were increased to 100 mM, growth with LNT and 3-FL was still lower compared to the other HMOs for serotype 6B strain 106.66 (Fig. S1B). Furthermore, we confirmed growth for all HMOs except 2′FL when Gal in a semi-defined medium (Lacks medium) was used for pre-growth for the serotype 2 strain D39 and its unencapsulated derivative R6 (Fig. S1C, D). Overall, although we had to increase the concentrations for some HMOs, we found that all the tested HMOs except LNT exhibited increased and continuous growth when pre-grown in Gal compared to Glc, for which there was a lag time corresponding to the moment Glc was likely to be totally consumed. This suggests that CCR is involved in the metabolism of these compounds.

### HMO metabolization depends on the presence of surface-associated exoglycosidases

BgaA, NanA, and StrH are important for the ability of *S. pneumoniae* to deglycosylate host glycans as a mechanism for colonization, evasion of the immune system, and/or pathogenesis^20,28–31^. Based on the structural similarities of HMOs with complex N-glycans, we hypothesized that these surface-associated exoglycosidases are essential for HMO metabolization (Fig. 1A).

We therefore included strains with *bgaA*, *nanA*, or *strH* deletions in the R6 and D39 backgrounds and compared growth with the wild-type (WT) strains (Fig. S2A–I). We found that *bgaA* (Fig. S2A and G) and *nanA* (Fig. S2E and H) were indeed essential for growth in LNnT and 6′SL, respectively. However, *bgaA* was identified as dispensable for growth using 3-FL and LNT as the sole C-source, respectively (Fig. S2B, C). As for 3′SL (Fig. S2F), deletion of *nanA* drastically reduced growth but did not eliminate it entirely. Growth results for LNnT suggested that *nanA* was irrelevant despite its upregulation compared to Lac (Fig. S2D and H). A growth defect was found for the *strH* KO strain in the medium containing LNnT (Fig. S2I). Taken together, BgaA, NanA, and StrH are essential for growth using the selected HMOs as carbon source. Since the expression of these exoglycosidase genes has been described to be controlled by CCR and their promoters contain *cre* sites^32^, this underscores the importance of growth conditions (such as pre-growth in Gal) in allowing the expression of these genes and facilitating the subsequent metabolism of HMOs.

**Fig. 2:**
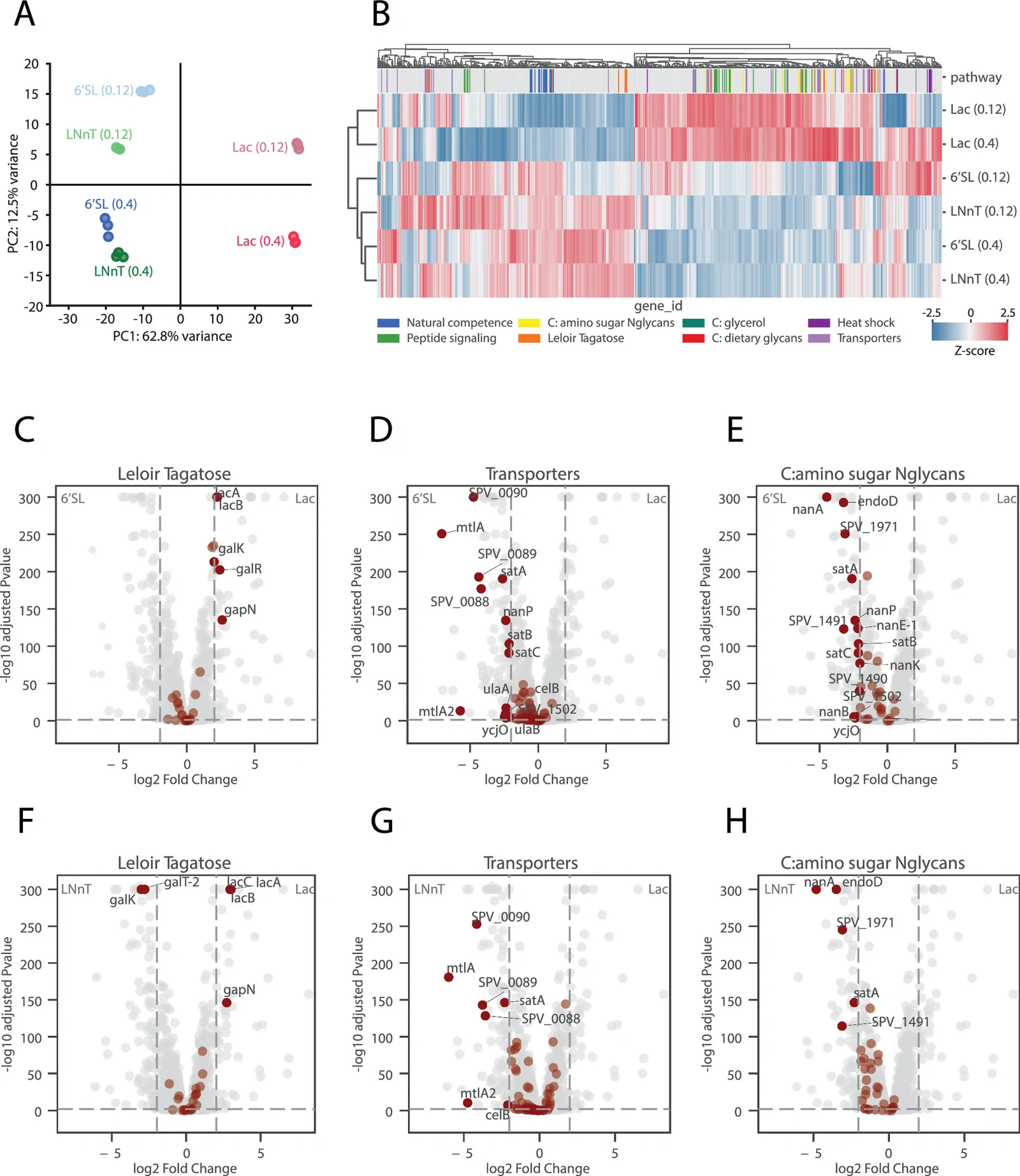
RNA-seq reveals expression differences for LNnT and 6′SL as compared to Lac. **A** Principal component analysis for three biological replicates of pneumococcal strain D39 grown in Lac, LNnT or 6′SL, based on rlog normalization^59^. Expression has been measured at two different time points (OD_600 nm_ = 0.12 and OD_600 nm_ = 0.4). **B** Heatmap is shown with varying *z*-scores of genes and their belonging to selected different pathways. **C–H** Volcano plots of differentially expressed genes (DEGs)  between D39 grown in Lac and 6′SL (**C**–**E**) or LNnT (**F**–**H**) up to OD_600 nm_ of 0.4 are illustrated. Genes of the Tagatose-6-phosphate/Leloir pathways (**C**, **F**) carbohydrate transporters (**D**, **G**) and amino sugar/N-gylcans (**E**, **H**) processing genes are coloured, and labelled if log_2_ fold changes were below −2.0 or above 2.0 (dotted lines). Differential enrichment was tested using DESeq2 (negative binomial generalized linear model with a Wald test) with two-tailed tests and *P*-values adjusted for false discovery rate.

### LNnT and 6′SL trigger specific transcriptional metabolic responses

We next aimed at revealing expression differences among media containing Lac or the two HMOs demonstrating the most robust bacterial growth to better understand the regulation of genes involved in metabolization beyond CCR. Thus, we investigated the transcriptome for strain D39 after growth in LNnT, 6′SL, and Lac for two different time points during growth (low optical density (OD) of 0.12 and high OD of 0.4) using RNA-seq. Based on the principal component analyses plot, we noted that (1) transcriptomes are reproducible within condition, (2) the greatest, consistent transcriptome difference is growth on Lac vs 6'SL or LNnT and (3) the second greatest transcriptome difference is in growth phase (i.e., different ODs) (Fig. 2A). Differentially regulated genes belonged largely to 8 different pathways (Fig. 2B–H, Supplementary Data 2, and Figs. S3–5). Importantly, we noted that the genes *galK* and *galR* from the Leloir pathway were highly increased in expression using 6′SL (as compared to Lac; Figs. 2C and S3 and Supplementary Data 3), but the opposite was found for *galK* if grown in LNnT (Figs. 2F and S3 and Supplementary Data 4). The Leloir pathway metabolizes Gal and it has been shown that GalR acts as a transcriptional activator of *galKT* in the presence of Gal in *S. pneumoniae*^33,34^. We also noted an increased expression for the tagatose-6-phosphate (T6P) pathway genes of the *lacI* operon, namely *lacA* and *lacB*, when grown in 6′SL as compared to Lac (Figs. 2C and S3 and Supplementary Data 3). Similar findings were observed when grown in LNnT as compared to Lac (Fig. 2F and Supplementary Data 4). It has been suggested that LacR acts as a transcriptional repressor of *lac* operon I, in the absence of Lac or Gal^35^ and that there is a complex interplay between the Leloir and T6P pathways, presumably mediated by intracellular concentrations of intermediates or end products of either pathway^34^. The latter may explain the expression patterns found for Leloir and T6P pathways when grown in LNnT. We also found (log_2_FC > 2.0) upregulation of various carbohydrate ABC transporters when grown in LNnT or 6′SL as compared to Lac (Fig. 2D and G).

Namely, the genes SPV_0088-90 and *satABC* were affected, which were previously described as transporting Gal, Mannose, N-acetylmannosamine and Sia^5,36^. Amongst PTS transporters, we found differences for Mannitol (*mtlA* and *mtlA-2*) and Cellobiose (*celBCD*) transporters (Fig. 2D and G). Regarding the genes of the amino sugar and N-glycan pathways, decreased expression of the exoglycosidases *nanA* and endo-β-N-acetylglucosaminidase (*endoD)* in Lac (as compared to LNnT and 6′SL) was noted (log_2_FC < −2.0) (Fig. 2E and H). The finding of *nanA* is consistent with the described function of *nanA* in cleaving Sia from 6′SL^37^. EndoD has been described to cleave the N-glycan core structure between two GlcNAc residues^15^, but neither 6′SL nor LNnT contains this compositional signature. We further identified that competence genes expression is upregulated when D39 is grown in 6′SL or LNnT as compared to Lac (Figs. S4 and S5). In addition, major differences were also noted for peptide signalling genes *vpoBCD* (upregulated in Lac) and *rtgBCD* (downregulated in Lac) at early and late growth phases (OD 0.12 versus OD 0.4) (Figs. S4 and S5 and Supplementary Data 5 and 6).

In summary, RNA seq revealed differentially expressed genes involved in the metabolism of LNnT and 6′SL, compared to Lac, which belong to transporters, exoglycosidases and amino sugar processing pathways. In addition to CCR, this shows that there is also HMO-specific regulation of the transcriptome.

### Genome-wide CRISPRi-seq identifies *lac* operon I and II (*lacEFG-2*) as essential for HMO metabolization

**Fig. 3:**
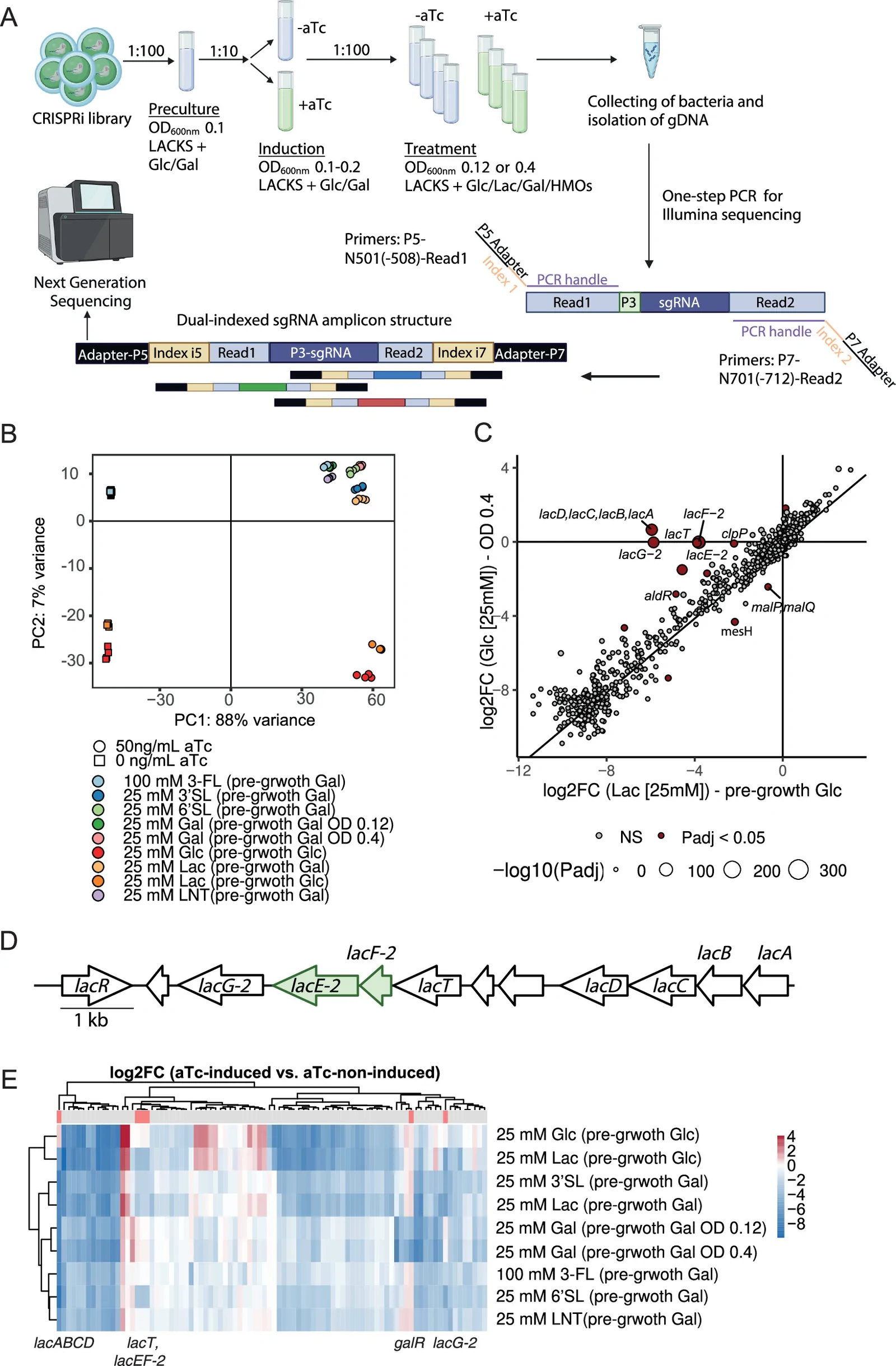
Anhydrotetracycline (aTc)-inducible, genome-wide CRISPR interference (CRISPRi) screen in *S. pneumoniae*. **A** Overview of the pooled fitness assay: the pooled CRISPRi library is grown under selective conditions, with (green) or without (blue) aTc. Cultures are kept in exponential growth phase by back-dilutions. PCR is performed from the extracted genomic DNA pool, sequenced (Illumina), and read counts are obtained. Changes in sgRNA abundance between induced and non-induced samples reflect gene fitness. Created in BioRender. Bacher, K. (2026) https://BioRender.com/mgxiepi. **B** Principal component analysis of genome-wide fitness profiles by CRISPRi-seq following aTc-induction on different carbon sources (Glc glucose, Gal galactose, Lac lactose, and the HMOs: 3-FL 3-fucosyllactose, 3′SL 3′-sialyllactose, 6′SL 6′-sialyllactose, and LNT Lacto-N-tetraose) of the Lacks medium showed reproducibility of replicates. For strains in Gal, two different set of replicates are shown (high and low OD). **C** Pair-wise CRISPRi-seq plot highlighting the essential genes of the *lacEF-2* operon in Lac metabolization. Two conditions, Glc compared to Lac, are shown. The other conditions are shown in Fig. S6. **D** Genetic organization of PTS-*lacEF-2* (adapted from ref. ^5^): *lacR* Lac phosphotransferase system repressor, *lacG-2* 6-phospho-beta-galactosidase, lacEF-2 PTS-Lac (EIIBC and EIIA), *lacT* transcription antiterminator, *lacD* tagatose 1,6-diphosphate aldolase, *lacC* tagatose-6-phosphate kinase, *lacA* and *lacB* Gal-6-phosphate isomerase subunit A and B. **E** Heatmap of all CRISPRi strains illustrating differentially enriched sgRNAs. For strains in Gal, two different sets of replicates are shown (high and low OD). In total, 98/1498 operons being differentially essential (∆|log_2_FC| >1, *P*_adj_ < 0.05) are indicated. Labelled genes: *lacABCD*, *lacT*, *lacEF-2*, *galR*, and *lacG-2*, showing differences between strains grown in Gal as compared to the HMOs and Lac. Differential enrichment was tested using DESeq2 (negative binomial generalized linear model with a Wald test) with two-tailed tests and *P*-values adjusted for false discovery rate.

CRISPRi-seq is a promising, unbiased genome-wide method for investigating gene fitness through different conditions. The strong advantage of this approach is the inducibility of the knockdown expression system that permits a resolution even on essential genes. Expression and CRISPRi data are showing different aspects of regulation and are complimentary. Therefore, we additionally undertook a systematic analysis of genes essential for HMO metabolization and performed a CRISPRi-seq screen using a previously constructed genome-wide knockdown library^38^ in Lacks medium supplemented with different C-sources (Fig. 3A). As D39 did not properly grow in CDM, a semi-defined medium (Lacks medium) has been chosen for the investigation of growth. In this approach, a catalytically inactive mutant of Cas9 (dead Cas9; aka dCas9) production is induced by anhydrotetracycline (aTc) in a pooled library of clones containing sgRNA specifically targeting each operon of the strain D39 (and R6) (HL23-1 and HL23-2; Table 1). The dCas9-sgRNA-DNA complex functions as a roadblock that stalls transcription elongation and thus generates a transcriptional knockdown mutant of the target gene/operon. Following deep sequencing of the library cultivated across conditions, we then quantified gene-wise fitness scores by comparing the corresponding sgRNA read counts in induced and non-induced replicates grown in media containing Glc, Lac, Gal, 6′SL, 3′SL, LNT, or 3-FL. As expected, CRISPRi induction explained most (PC1: 88%) of the variance between samples in a dimensional reduction analysis (Fig. 3B). Importantly, we included two different sets of replicates for Gal which were grown to an OD of 0.12 and 0.4, respectively, to adjust for the fact that there is a moderate growth for some HMOs (OD_max_ of around 0.2 for 3-FL and LNT) or very efficient growth for others (OD_max_ of around 0.8 for Lac, 3′SL, and 6′SL) (Fig. 3B). We then compared the fitness scores between cells grown in Glc/Gal vs HMOs and visualized the data as interaction plots (Figs. 3C and S6). Most prominently, knockdown expression of the *lacEFG-2* genes (encoded within *lac* operon II—PTS-*lacEFG-2*^5^; Fig. 3D) resulted in a fitness loss during growth in Lac, 6′SL, and 3′SL, as compared to Glc (Figs. 3C and S6A) or Gal (Fig. S6B–D). However, LNT and 3-FL only showed *lacG-2* (encoding a 6-phosphate-beta-galactosidase) but not the PTS transporter genes *lacEF-2* as being differentially essential (Fig. S6E, F). Although *lacG-2* is essential in all HMO-based media, the findings do not indicate essential transporters that could be involved in the uptake of 3-FL and LNT in contrast to *lacE-2* for the other tested HMOs. Overall, we observed condition-specific fitness profiles among all HMOs, Lac and Gal, with 98/1498 operons being differentially essential between at least 2 conditions (∆|log_2_FC| >1, *P*_adj_ < 0.05) (Fig. 3E and Supplementary Data 7).

We next performed identical experiments using the R6 strain and, importantly, we found the same essentiality patterns for the *lac* operon II (*lacEFG-2*) (Figs. S7 and S8 and Supplementary Data 8). This underlines the robustness and reproducibility of our findings. We then created and tested individual gene deletion strains, D39Δ*lacG-2* and serotype 7 F strain 208.41Δ*lacG-2*, to validate the screen (Tables 1 and 2). The latter was chosen to verify the results, using a clinical strain that could be transformed efficiently and that can metabolize Sia as D39 is unable to ferment Sia due to a frame shift in the neuraminate lyase of the *nanAB* locus^36^. As expected, we found that the D39Δ*lacG-2* and 208.41Δ*lacG-2* strains showed growth delays and/or decreased maximal OD when cultivated with Lac and all HMOs (Fig. S9).

**Table 2:** Primers used for strain construction

Name	Sequence
CRISPRi library_*tetR*_f	5′-GTGAAGTTATGAACATCATCGG-3′
CRISPRi library_*tetR*_r	5′-GGTTCGCAAGCCATGG-3′
CRISPRi library_*dCas9*_f	5′-ATTCCTTCTTAACGCCCCAAGTTC-3′
CRISPRi library_*dCas9*_r	5′-GTCTTCTTTTTTACCTTTAGTAACTACTAATCCTGCAC-3′
DAM406^61^	5′-TCT ATG CCT ATT CCA GAG GAA ATG GAT-3′
DAM347^61^	5′-CCG AAT TCT AGG TAC TAA AAC AAT TCA TCC AGT AA-3′
D39/R6_Δ*lacG-2*_HA1 f	5′-ATGACAAAAACACTTCCAAAAGACTTTATTTTTG-3′
D39/R6_Δ*lacG-2*_HA1 r	5′-ACACGGATCCAAAATGTTCGATATTTTC-3′
D39/R6_Δ*lacG-2*_HA2 f	5′-ATCGGAATTCAAGAAGATTTACATCAC-3′
D39/R6/208.41_Δ*lacG-2*_HA2 r	5′-CTAGTCTATAATCTGAGTTTCCGCTACTTTC-3′
208.41_Δ*lacG-2*_HA1 f	5′-ATGACAAAAACACTTCCAAAAGACTTTATTTTCG-3′
208.41_Δ*lacG-2*_HA1 r	5′-ACACGGATCCAAAATGTTCGATATTTTC-3′
208.41_Δ*lacG-2*_HA2 f	5′- ATCGGAATTCTAAGAAAATCTACATCAC-3′

*HA* homologous arm.

In order to verify the non-differences, we then compared the growth of WT and the *lacG-2* deletion strains in Lacks containing Gal. Growth was comparable when comparing the WT to the Δ*lacG-2*-strains in Gal (Fig. S10A).

In addition, we also tested a Δ*lacE-2* null mutant (FP382) in an R6-like background using the same growth conditions (Figs. S10B and S11A–F). Again, the findings of the CRISPRi-seq screen were confirmed. The WT strain, compared to the gene deletion strains, showed only a minor difference in LNT and 3-FL, while growth of the deletion mutants was impaired on Lac, 3′SL, 6′SL, and LNnT as sole C-sources. Therefore, the findings from the CRISPRi-seq could clearly be validated using loss of function mutant strains.

### The cellobiose-specific phosphotransferase system (PTS) does not play a major role in HMO metabolization

Of note, growth of the Δ*lacE-2* null mutant (FP382; Table 1) and the Δ*lacG-2*-strain was not completely abolished as we observed delayed growth for Lac, 3′SL, 6′SL, and LNnT, suggesting a compensatory mechanism (Fig. S11A, C–E). For the *Lactococcus lactis* laboratory strain MG1363, it has been suggested that increased expression of *celB*, encoding a putative cellobiose-specific PTS IIC component, might be relevant for the transport of Lac^39^. Indeed, we found the genes *celB* and *celD* to be upregulated when grown with LNnT and 6′SL (Fig. 2D, G), and they have been described to be under CCR due to the presence of *cre* sites^39^. Therefore, we tested the growth of the Δ*celB* (FP415) and the Δ*celD* (FP435) null mutant (Fig. S12). However, no growth deficit was observed for the Δ*celB* (FP415) and Δ*celD* (FP435) null mutants. This suggests that *celB* and *celD* do not seem important for growth on HMOs in contrast to *lacE-2*.

### Quantification of extracellular products reveals an increase in Gal and mixed fermentation profiles for LNnT

Next, we aimed to better understand the degree of mixed acid fermentation and whether certain monosaccharides or oligosaccharides accumulate extracellularly during the growth of *S. pneumoniae* in HMOs and Lac. To investigate this, we recorded ^1^H-NMR spectra of the chemically defined medium (CDM) from post-culture samples to quantify the decreasing substrates and increasing metabolic products at different growth stages. This was done by quantifying the concentrations derived from the α-Gal H1 (free Gal) and β-Gal H1 (Gal from LNnT) resonances from the ^1^H-NMR spectra.

**Fig. 4:**
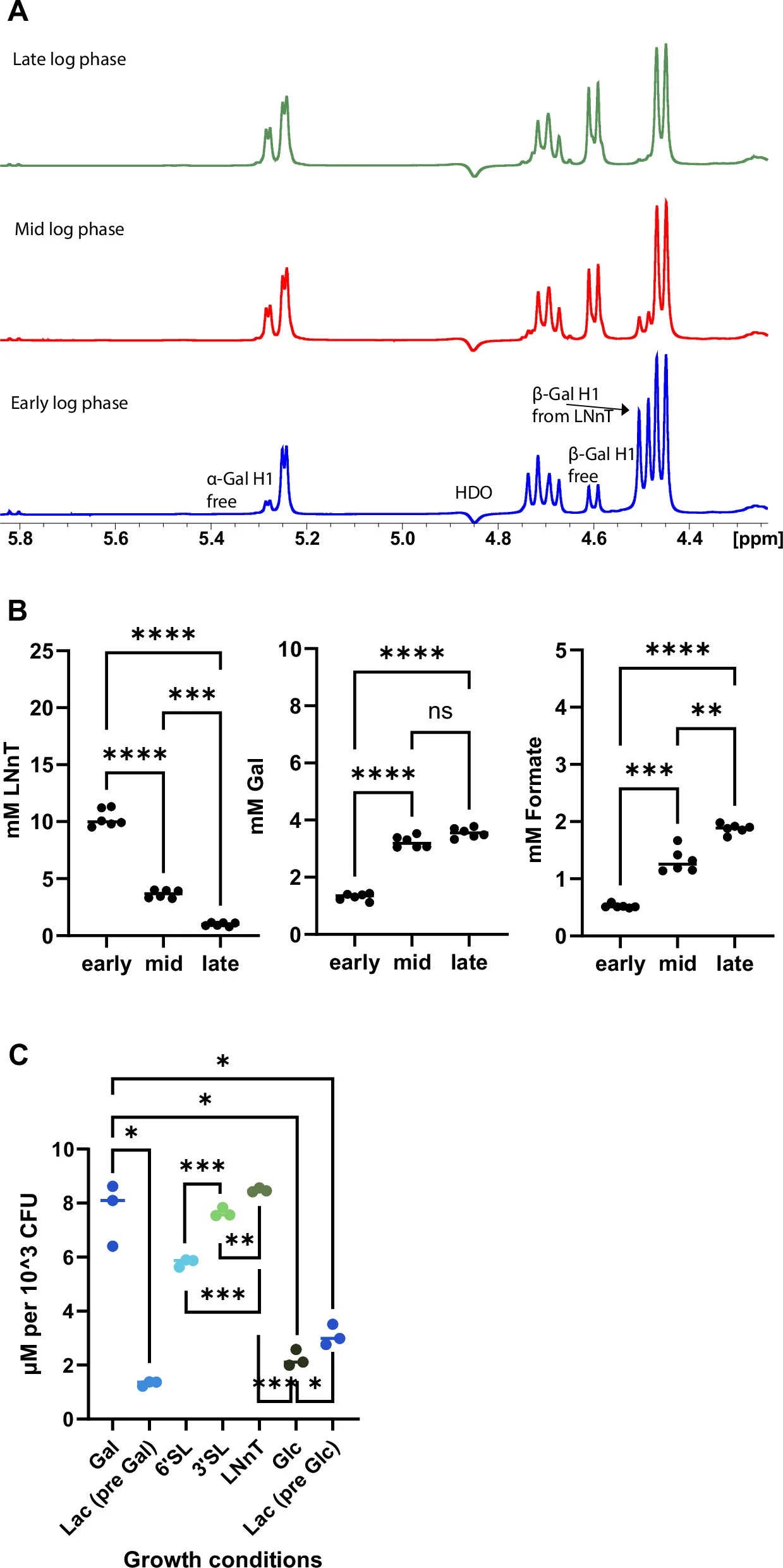
^1^H-NMR spectra of *S. pneumoniae* grown in CDM-LNnT and calculations of concentrations of LNnT, Gal, Formate, and H_2_O_2_ during three growth phases. ^1^H-NMR spectra were received for extracellular metabolites after *S. pneumoniae* culturing in LNnT until early, mid and late log phase (**A**). Gal was quantified using the anomeric proton signal of α-Gal H1 at 5.2 ppm. Residual LNnT was monitored using the β-Gal H1 signal at approximately 4.5 ppm and formate was quantified using its proton resonance at 8.4 ppm. With increasing OD, LNnT metabolism leads to increased and decreased levels of Gal  and LNnT, respectively (**B**). Metabolite quantification was done from six biological replicates and statistical significance was assessed using one-way ANOVA Tukey’s multiple comparisons test, ****p < 0.0001, ***p = 0.0002, **p = 0.001 (**B**). Subsequent H_2_O_2_ quantification from three independent experiments using a specific kit revealed differences for the different HMOs indicating mixed acid and homolactic fermentation, statistical significance was assessed using one-way ANOVA Tukey’s multiple comparisons test. ns not significant (*p* ≥ 0.05), * 0.01 < *p* < 0.05, ** 0.001 < *p* < 0.01, *** 0.0001 < *p* < 0.001, and **** *p* < 0.0001 (**C**).

We found decreasing Gal from LNnT and increasing free Gal concentrations over time when grown in LNnT (Fig. 4A, B), which is in accordance with the upregulation of Leloir pathway genes found in our transcriptomic data (Fig. 2). In addition, both fermentation products, formate and lactate, increased in concentration with increasing OD (Fig. 4B). However, we did not find an increase in “free” Lac during growth. Increasing Gal was also found when *S. pneumoniae* was grown in Lac (Fig. S13). Finally, 3-FL growth analysis suggested that the substrate (3-FL) is hardly consumed (Fig. 1), and we therefore hypothesize that the observed minor growth in Lacks (Fig. S1C, D) could alternatively result from the metabolization of contaminating metabolites rather than actual 3-FL (Fig. S14).

It has been demonstrated that Glc supplementation results in the accumulation of lactate, whereas the presence of Gal results in higher levels of formate due to a switch from homolactic to mixed acid fermentation^40^. In addition, it has been shown that loss of CcpA also causes a shift towards a more mixed acid fermentation and that the H_2_O_2_ concentration is circa sevenfold enhanced as compared to the WT D39 strain^8^. In our study, quantification of H_2_O_2_ done by using a specific assay showed similar concentrations when grown in LNnT as compared to Gal, while the opposite was found for Lac (Fig. 4C). Based on the above transcriptomic data, differential gene expression (Lac versus LNnT) at OD 0.4 showed Log_2_FC values of −1.4 for the pyruvate pathway genes *pfl* and −1.4 for *adh* (Supplementary Data 3), which is expected considering the enhanced H_2_O_2_ concentration and suggests mixed acid fermentation.

### 2′FL inhibits the adherence of *S. pneumoniae* to Detroit and primary nasal epithelial cells

**Fig. 5:**
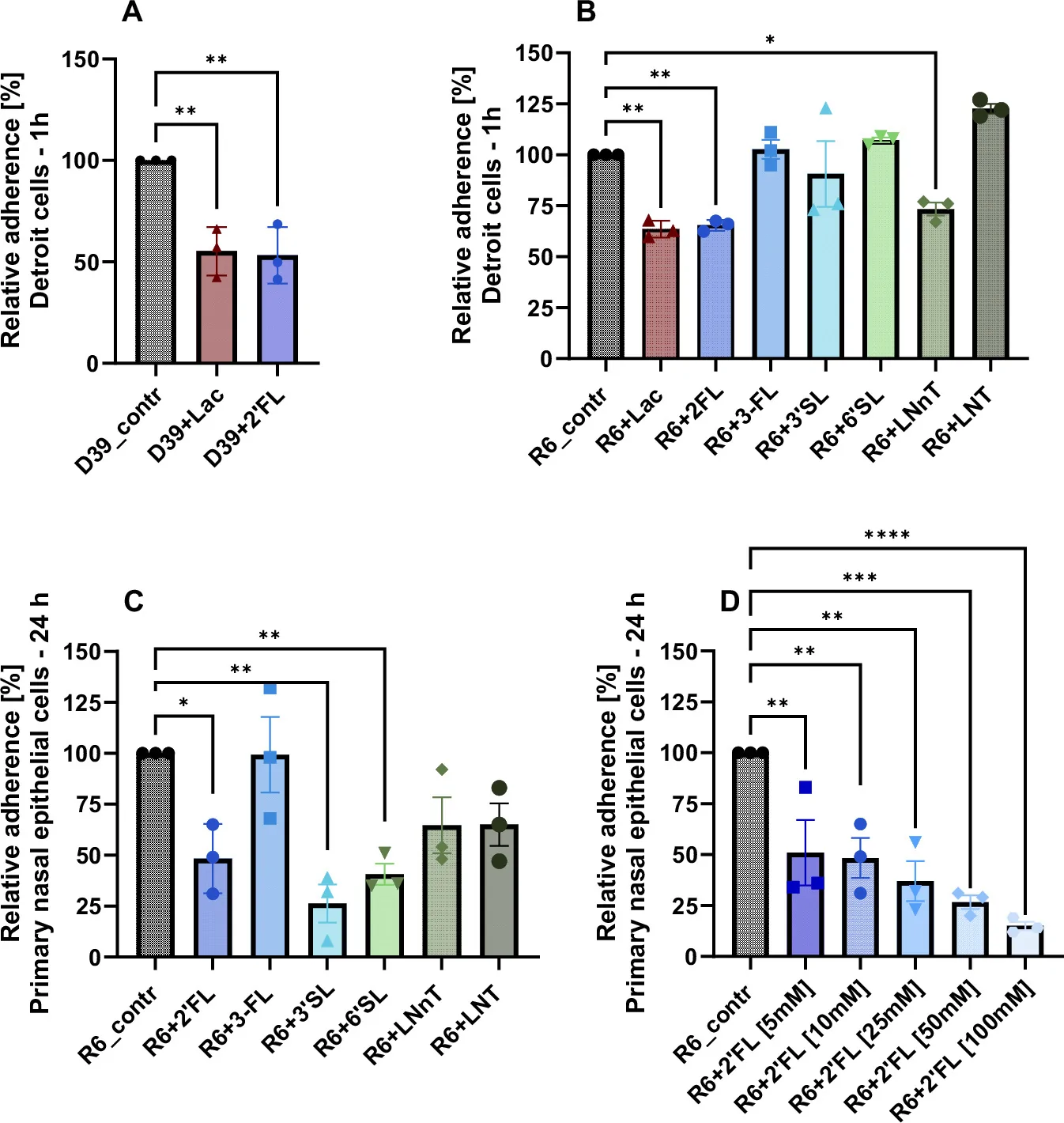
2′-fucosyllactose (2′FL) reduces pneumococcal adherence in a concentration-dependent manner. Detroit 562 cells, 10 mM of lactose (Lac) and 10 mM of each of the human milk oligosaccharides (HMOs: 2′FL, 3-fucosyllactose (3-FL), 3′-sialyllactose (3′SL), 6′-sialyllactose (6′SL), Lacto-N-neotetraose (LNnT), and Lacto-N-tetraose (LNT)) were used to compare their potential for adherence reduction of wild type D39 (**A**) and the non-encapsulated R6 strain (**B**). The control (ctrl.) did not contain additional Lac or HMOs during adherence. The air-liquid interface (ALI) cell culturing method was used to further investigate the potential of HMOs to decrease pneumococcal (R6) adherence to primary nasal epithelial cells (**C**). Different concentrations of 2′FL were investigated in an ALI culturing model with primary nasal epithelial cells from three human donors (**D**). Adherence was measured by counting bacteria (R6) on CSBA plates after 1 h (Detroit cells) or 24 h (primary nasal epithelial cells) of incubation, and the CFU/mL was normalized to the original inoculum. In (**A**, **B**), the averages ± SEM of three independent experiments, each performed in three technical replicates from Detroit cells, are shown. In (**C**, **D**), the averages ± SEM are plotted for each concentration from data using primary cells of three independent experiments. Again, statistical significance was assessed using one-way ANOVA Dunnett’s multiple comparisons test: ns not significant (*p* ≥ 0.05), * 0.01 < *p* < 0.05, ** 0.001 < *p* < 0.01, *** 0.0001 < *p* < 0.001, and **** *p* < 0.0001.

Complex N-glycans display similar sugar moieties as HMOs and act as both food sources and “docking stations” for *S. pneumoniae*, underlining the fine balance between the benefits of carbohydrate receptor binding (adherence) and deglycosylation (food source)^15^. Thus, we further hypothesized that the presence of HMOs leads to inhibition of pneumococcal adherence to epithelial cells. Encapsulated strains are known to be significantly less adhesive compared to non-encapsulated strains^41^. Thus, adherence assays using both D39 and the non-encapsulated strain R6 on Detroit 562 cells in the presence of either Lac or single HMOs [10 mM] were performed. However, we found that both Lac and 2′FL decreased adherence to Detroit 562 nasopharyngeal cells after 1 h, but there was no significant difference between Lac and 2′FL (Fig. 5A, B). We then tested the effects of the other HMOs on R6 adherence to Detroit cells (Fig. 5B). The only other HMO that showed a significant reduction in adherence to Detroit cells was LNnT (*p* < 0.05), which also is in line with previous observations^42^.

To further investigate the effect of HMOs (used concentration of [10 mM]) on cells in a more physiological environment, an air-liquid interface (ALI) cell culture model using primary nasal epithelial cells was utilised (Fig. 5C, D). Again, 2′FL showed a significant decrease in adherence after 24 h to primary nasal epithelial cells (*p* < 0.01). Interestingly, in the ALI cell culture model, 3′SL and 6′SL, but not LNnT, showed a significant reduction in adherence after 24 h. As 2′FL was the only HMO that showed a significant decrease in adherence in both culturing models with different adherence durations, we aimed to further investigate 2′FL by using different concentrations. 2′FL could significantly decrease adherence in a concentration-dependent manner in the ALI model (Fig. 5D). Taken together, 2′FL inhibits adherence similarly to Lac, but, in contrast to Lac, 2′FL cannot be metabolized. Therefore, we hypothesized that 2′FL might be better suited to prevent adherence.

### Metabolization and adherence inhibition of Lac and 2′FL in adult mice

**Fig. 6:**
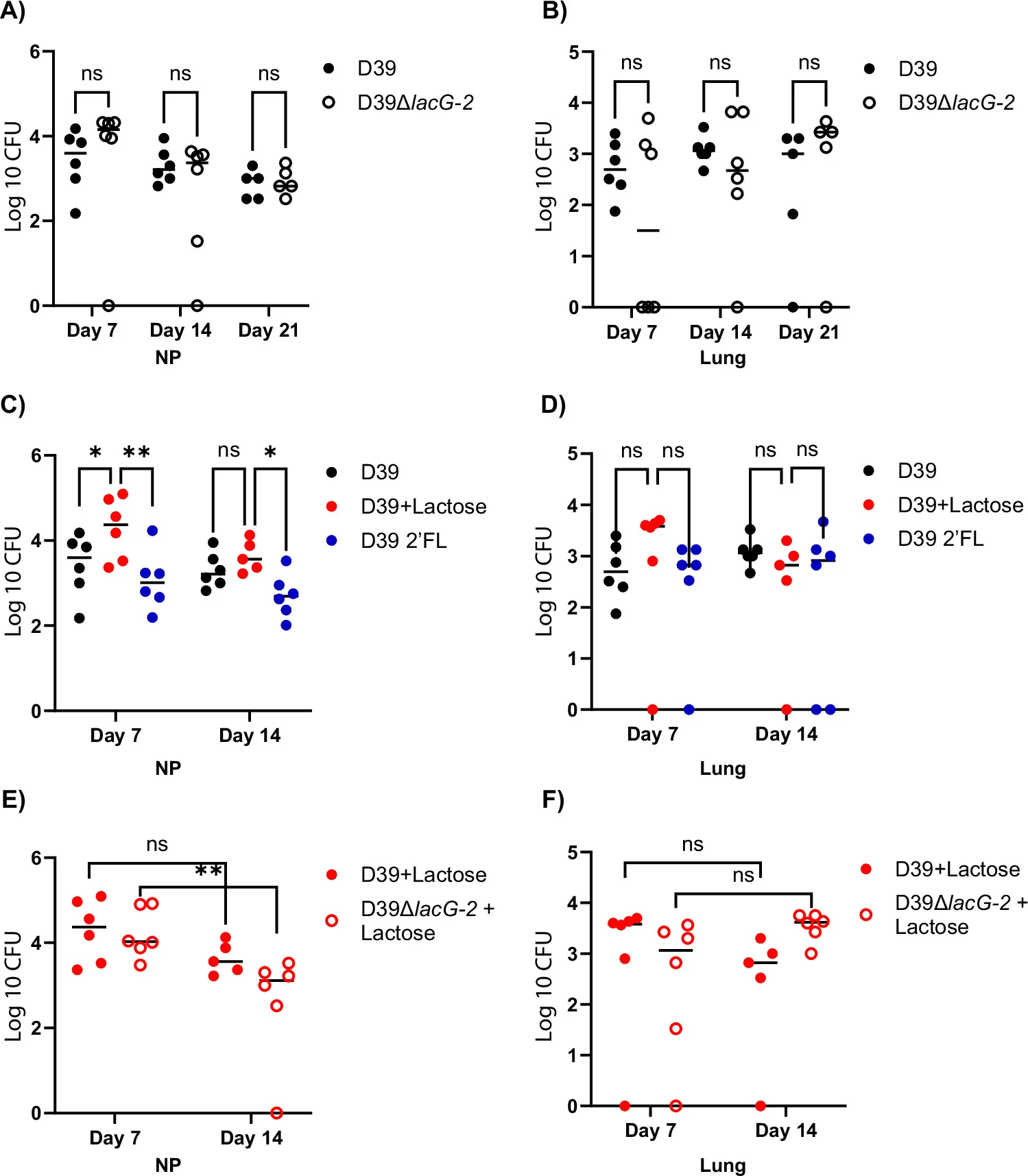
Inhibition of D39 and D39Δ*lacG-2* adherence using lactose (Lac) and 2′-fucosyllactose (2′FL) in adult mice. Shown are the results of long-term carriage experiments (cfu counts after 7, 14, and 21 days) in an in vivo murine model using the D39 and D39Δ*lacG-2* strains, ns not significant (*p* ≥ 0.05) (**A**, **B**). In addition, the inhibitory effects of Lac and 2′FL were tested in the long-term carriage experiments using WT D39 (cfu counts after 7 and 14 days), * *p* = 0.0465 (D39vs. D36 + Lac), * *p* = 0.0425 (D39 + Lac vs D39 + 2′FL), ** *p* = 0.0040 (**C**, **D**). Adherence inhibition of Lac was additionally investigated using both the D39 and D39Δ*lacG-2* strains, ** *p* = 0.0076 (**E**, **F**). Individual mice were considered as the experimental unit. Statistical significance was assessed using two-way ANOVA Šídák’s multiple comparison test.

Finally, we aimed to investigate whether what was observed in vitro could be reproduced in vivo. Thus, we next conducted long-term nasopharyngeal carriage experiments using an in vivo murine model. Using the D39 and D39Δ*lacG-2* strains for inoculation, we first found that the deletion of *lacG-2* did not reduce the carriage density after 7-, 14-, and 21-days post-infection (Fig. 6A, B). We next aimed at testing if 2′FL is better suited to prevent pneumococcal carriage in vivo as compared to Lac, as we hypothesized that non- or slowly metabolizable HMOs (e.g., 2′FL) could provide increased adherence inhibition compared to Lac. For this, Lac or 2′FL were administered intranasally to mice at the point of infection and subsequently on days 4 and 10 post-infection. Decreased colony-forming units (cfu) in the nasopharynx were observed for 2′FL-treated mice, relative to those treated with Lac (Fig. 6C). Lac increased bacterial density in the nasopharynx at day 7 post-infection, relative to the untreated control group (Fig. 6C). No differences were seen for the lung samples (Fig. 6D). Interestingly, a slight decrease in adherence was noted for the D39Δ*lacG-2* strain if co-inoculated with Lac in the nasopharynx but not the lung samples (Fig. 6E, F). We conclude that the inhibitory effects of Lac on adherence may be more pronounced in strains unable to metabolize Lac, as shown for the D39Δ*lacG-2* strain.

## Discussion

Studies investigating the metabolism of monosaccharides in *S. pneumoniae* showing that there is redundancy in terms of substrate specificity of the carbohydrate transporters^5^ and that Glc is the preferred C-source^43^. However, compared to monosaccharides, the metabolization of HMOs is less well described, which has been the focus of our study. HMOs are important as they are found to be absorbed into the bloodstream of breastfed infants, suggesting their systemic availability^24^. In addition, some HMOs have been suggested to impair adherence, which make them interesting candidates for the development of new prebiotic strategies to prevent pneumococcal infections^44,45^. Therefore, we sought to determine if (1) HMOs are primarily cleaved by extracellular proteins and then imported as monosaccharides, or (2) they are imported as OS, with cleavage happening intracellularly. We then (3) tested the hypothesis if non-metabolizable HMOs are particularly suited to inhibit pneumococcal adherence. We investigated the metabolization of different HMOs and performed expression analyses, CRISPRi-seq screens and adherence assays.

First, we found that growth using the tested HMOs is under CCR, as pre-growth with Gal increased the growth rates. Also, we confirmed previous findings that exoglycosidases like NanA, BgaA, and StrH are essential for growth^8,32,46,47^. Considering promotors of the exoglycosidase genes contain *cre* sites^8^, this suggests that *S. pneumoniae* should not be grown in Glc containing media as genes would not be properly expressed^36^, as confirmed by our growth data confirmed this suggestion.

However, a study in *L. lactis* revealed that the PTS-*lacFE* is necessary for Gal metabolism, thus implicating this transporter in Gal uptake^48^. In addition, dephosphorylation by an unknown phosphatase has been suggested during Lac uptake and further catabolism in *L. lactis* MG1363^39^. It has also been described that ManNAc (used as a substitute to Sia) has a positive effect on the induction of *lacA* and *galK* promoters irrespective of Gal concentration, and growth on Gal, consistent with a switch to preferentially process ManNAc in *S. pneumoniae*^49^. Our transcriptomic data showed that the genes of *lac* operon I (*lacABCD*) are upregulated in Lac as compared to LNnT and 6′SL, indicating that the genes of the T6P pathway play a major role for Lac but not necessarily for LNnT and 6′SL.

However, the expression of ABC transporter genes SPV_0088-90, which has been hypothesized to transport Gal^5^, was found to be upregulated for the two tested HMOs, as compared to Lac. In addition, in the case of LNnT, the Leloir pathway genes were also upregulated, suggesting metabolization of Gal rather than Gal6P, as “free” Gal is a prerequisite for its usage via the Leloir pathway^49^. Taken together, the transcriptomic data showed that there is an additional regulation for LNnT, 6′SL, and Lac metabolization beyond CCR.

Considering that HMOs contain Lac at their reducing end, it may not be surprising that the genes of the *lac* operon II (*lacEFG-2*) were identified as essential in our CRISPRi screens for all tested HMOs. Of note, we found that using Lacks medium in our experiments allowed a very clear and clean identification of essential genes using different C-sources in our CRISPRi-seq experiments. While the usage of Lacks medium with one defined C-source cannot be directly compared to an in vivo situation, it allows for a defined investigation of differentially essential genes. Notably, only *lacG-2*, but none of the transporter genes of the lac operon II (*lacEFG-2*), were found to be essential for the metabolization of LNT and 3-FL. This situation might be explained by transporter redundancy. In this case, knocking down only one transporter gene with a single sgRNA would not affect growth/fitness. Future work, for instance, applying dual CRISPRi-seq^50^, may provide insights into transporter redundancy. However, considering that our ^1^H-NMR data indicates that 3-FL is not consumed, we hypothesize that the observed minor growth in the presence of high concentrations could also be due to “contaminating” metabolites rather than actual 3-FL. As for LNT, it has been hypothesized that BgaC, and not BgaA, is responsible for its metabolization, as it has been shown that BgaC encodes a BgaA that specifically hydrolyses a beta-1,3-linked Gal to GlcNAc^31^. In our study, growth in LNT was low and we perhaps did not select the right growth conditions allowing BgaC to function properly, which could have favored the growth. Therefore, for our chosen growth conditions, we hypothesize a similar scenario for LNT as 3-FL, i.e., this situation might be explained by transporter redundancy or the observed minor growth in the presence of high concentrations of LNT could also be due to “contaminating” metabolites rather than actual LNT.

**Fig. 7:**
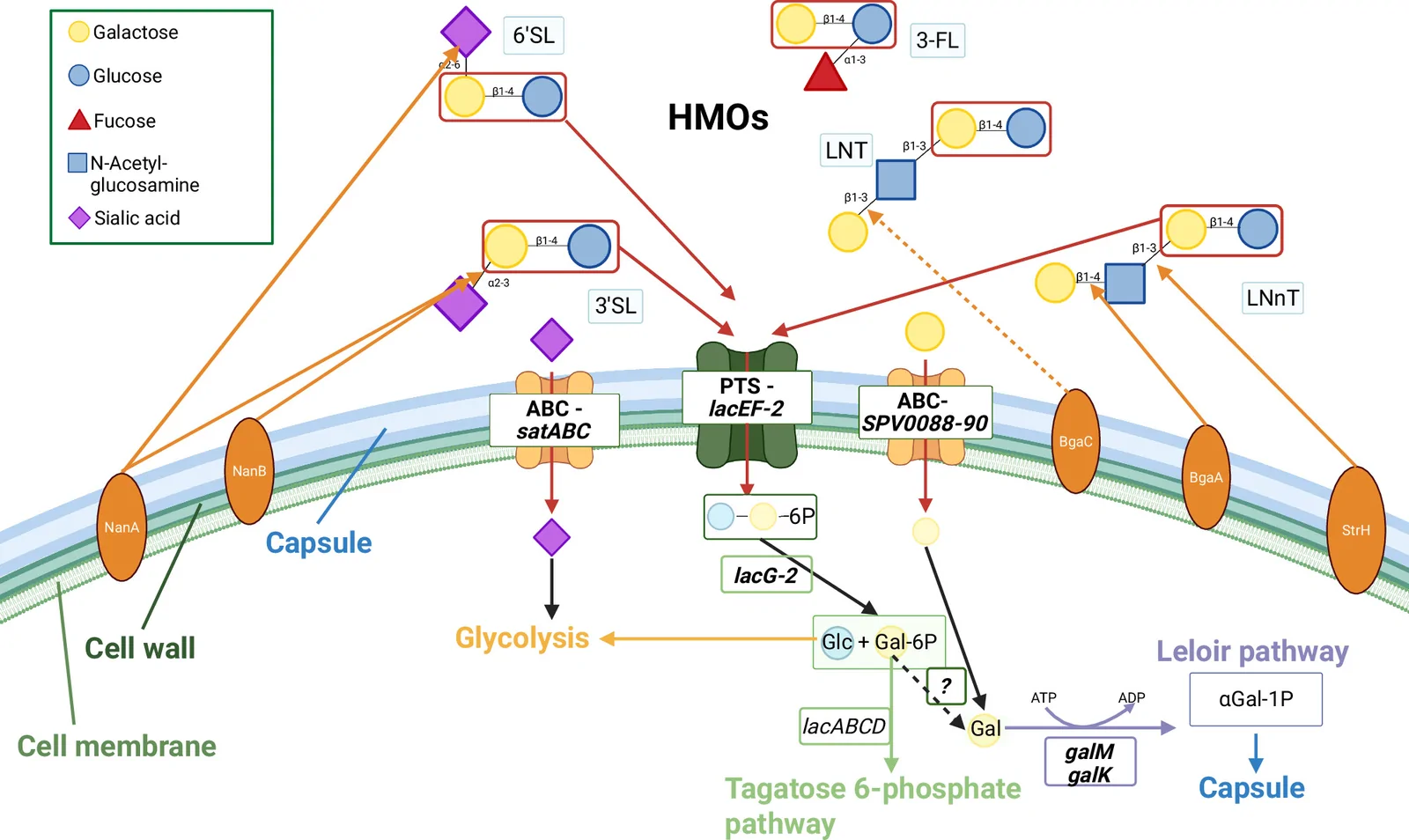
Proposed mechanisms of the relevant enzymes involved in human milk oligosaccharides (HMO) metabolization. Metabolization and transport of human milk oligosaccharides (HMOs): 3-FL 3-fucosyllactose, 3′SL 3′-sialyllactose, 6′SL 6′-sialyllactose, LNnT Lacto-N-neotetraose, and LNT Lacto-N-tetraose. PTS-*lacEF-2* PTS-Lac (EIIBC and EIIA), ABC-*satABC* N-Acetylneuraminic acid transporter, ABC-*SPV0088-90* galactose transporter, NanA neuraminidase A, NanB neuraminidase B, BgaA beta-galactosidase A, BgaC beta-galactosidase C, beta-N-acetylglucosaminidase (StrH), Lac-6P Lactose-6-phosphate, LacNAc-6P N-acetyl-lactoseamine-6-phosphate, *lacG-2* 6-phosphate-beta-galactosidase, Glc glucose, Gal galactose, GlcNAc N-acetyl-glucosamine, *lacAB* Gal-6-phosphate isomerase subunit A and B, *galM* aldolase 1-epimerase, *galK* galactosinkinase, ATP adenosine-tri-phosphate, ADP adenosine-di-phosphate, αGal-1P Galactose-1-phosphate. Orange dashed line indicates a hypothesized cleavage of BgaC^31^, which could not be confirmed in this study. Regarding metabolization and transport of LNT and 3-FL, two different hypotheses are discussed (see text for details). 2′FL is omitted as it is not metabolizable. Black dashed line indicates a hypothesized as-yet unidentified phosphatase responsible for dephosphorylating Gal-6-phosphate into “free” Gal. The lactose subunits are indicated for all the tested HMOs. Created in BioRender. Hilty, M. (2026) https://BioRender.com/xxwrpyd.

Importantly, as derived from our follow-up growth experiments using WT and gene deletion mutants, *lacG-2* was also identified as being essential in the clinical strain 208.41 for all tested HMOs, despite being able to metabolize Sia, in contrast to D39, which has a frameshift mutation in the neuraminate lyase^36^. Taking our growth, transcriptome, CRISPRi-seq, and metabolomics results together, we propose the following metabolic pathway for 3′SL, 6′SL and LNnT metabolization (Fig. 7). The neuraminidases (NanA and NanB) and BgaA cleave off sugar moieties extracellularly so that Lac, N-Acetyllactosamine (LacNAc), or unknown Lac-containing intermediate compounds can be imported into the cell via the PTS-*lacEF-2* transporter. After transport, the Lac6P is metabolized by LacG-2. Probably, an as-yet unidentified phosphatase is subsequently responsible for dephosphorylating Gal-6-phosphate into “free” Gal, which is then again phosphorylated via aldolase 1-epimerase (GalM) and a galactokinase (GalK) before entering the Leloir pathway. Also, transcriptome and NMR data suggest that “free” Gal and probably the aminosugars GlcNAc and Sia may also be imported by the ABC transporters SPV_0088-90 and SatABC, respectively (Fig. 7).

*S. pneumoniae* carbohydrate-mediated adherence has been investigated previously^44,45^. Specifically, sialylated OS have been suggested to impair adherence, but the role of fucosylated OS (e.g., 2′FL) is relatively unknown^44,45^. A recent study revealed that inhibitors of BgaA activity reduced pneumococcal growth and increased opsonophagocytic killing in a BgaA-dependent manner, whereas LacNAc and Lac were highlighted as potent inhibitors^30^. In more detail, it was suggested that two newly identified non-enzymatic carbohydrate-binding modules of BgaA mediate adherence to the host cell surface displayed Lac or N-acetyllactosamine. We confirmed that Lac inhibited adherence in both Detroit and primary cells. In addition, some of the tested HMOs were also found to inhibit cell adherence, but only 2′FL was found to inhibit both adherence to Detroit (after 1 h) and primary cells (after 24 h).

A recent genome-wide association study revealed that variants within *lacE-2* were strongly associated with carriage isolates from children with pneumonia compared to those from healthy community-based children^51^. In our mouse models, we did not  test a *lacE-2* mutant strain but did not find that *lacG-2* was important for carriage. This could indicate that OS containing Lac units are not metabolized during carriage in adult mice, perhaps due to limited availability. Therefore, we retested carriage in adult mice receiving Lac or 2′FL. We hypothesized that Lac, unlike 2′FL, can be metabolized by *S. pneumoniae* and that, therefore, the usage of 2′FL for the prevention of pneumococcal colonization might be preferred. Our results suggested that this may indeed be the case.

In summary, this study discloses the regulatory pathways and essential genes for the metabolization of the tested HMOs. In addition, it suggests the usage of 2′FL as a potential inhibitor for pneumococcal adherence. Understanding the metabolic flux of HMOs is important, as the prevention of HMO metabolization or inhibition of pneumococcal adherence using non-metabolizable HMOs might be interesting therapeutic avenues to pursue in the future.

## Methods

### Bacterial strains and growth conditions

All bacterial strains used in this study are described in Table 1. *S. pneumoniae* strains were streaked onto Columbia agar plates (CSBA) supplemented with 5% sheep blood and grown overnight at 37 °C and in an atmosphere of 5% CO_2_. When necessary for transformation, the media were supplemented with the following antibiotics: tetracycline (Merck) 5 mg/mL, gentamycin (Merck) 40 mg/mL, spectinomycin (Merck) 100 mg/mL, or kanamycin (Merck) 500 µg/mL.

### Growth assays

The liquid media used in this study were Lacks medium^52^ and CDM^53^. Bacteria were incubated at 37 °C and 5% CO_2_. Measuring bacterial growth was conducted with OD at a wavelength of 600 nm (OD_600 nm_) using a Helios Epsilon UV–visible spectrophotometer (Thermo Scientific) with an adapter that allows direct measurement in the culture tubes. For the first growth step in liquid media (pre-growth), the media was supplemented with either 20 mM Glc, Gal, or Lac till the desired OD_600 nm_ of 0.5 was reached. Centrifugation (2 × 3000 × *g* for 5 min) and washing steps (Lacks or CDM without C-source was used for washing) were conducted following the pre-growth. The bacterial suspension was then added (1:25) to the liquid media (Lacks or CDM) with different sugar concentrations (mono- (Glc and Gal), di- (Lac), and purified HMOs (2′FL, 3-FL, LNnT, LNT, 6′SL, and 3′SL (Supplementary Data 1)). The second liquid culturing step was measured again in the Helios Epsilon UV–visible spectrophotometer (for growth measurements in tubes), and for growth in plates, either the VICTOR® NIVO^TM^ Plate Reader or the BioTek Cytation 5 with shaking (5–30 s at 600 rpm) before each measurement was used. The cultures were grown, and OD was measured every 30 min for up to 10–20 h.

### RNA-seq of D39 in Lac, LNnT, and 6′SL

Bacteria were grown in LACKS supplemented with 25 mM C-source (Lac, LNnT, or 6′SL) until early (OD_600 nm_ of 0.12) or mid-log phase (OD_600 nm_ of 0.4), then RNA transcription was stopped with RNAprotect® bacterial reagent (QIAGEN, cat. no. 76506) and stored at −20 °C overnight. RNA extraction of three replicas was done as previously described in ref. ^32^.

The quantity and quality of the purified total RNA were assessed using a Thermo Fisher Scientific Qubit 4.0 fluorometer with the Qubit RNA BR & HS Assay Kits (Thermo Fisher Scientific, Q10211 & Q32855) and an Advanced Analytical Fragment Analyzer System using a Fragment Analyzer RNA Kit (Agilent, DNF-471), respectively. Thereafter, 100 ng of input RNA was depleted of ribosomal RNA using an Illumina Ribo-Zero plus rRNA Depletion Kit (Illumina, 20037135). Ribodepleted RNA was made into cDNA libraries using a TruSeq Stranded Total RNA Human/Mouse/Rat Kit (Illumina, 20020597) with IDT for Illumina TruSeq RNA UD Indexes (Illumina, 20022371). This was done according to the reference guide entitled “TruSeq Stranded Total RNA with Illumina Ribo-Zero Plus rRNA Depletion (Illumina, Document # 1000000092426 v01). The resulting cDNA libraries were evaluated using a Thermo Fisher Scientific Qubit 4.0 fluorometer with the Qubit dsDNA HS Assay Kit (Thermo Fisher Scientific, Q32854) and an Agilent Fragment Analyzer (Agilent) with a HS NGS Fragment Kit (Agilent, DNF-474), respectively. The libraries were equimolar pooled and sequenced with a loading concentration of 750 pM, paired-end and dual indexed, using an Illumina NextSeq 1000/2000 P2 XLEAP-SBS Reagent kit (Illumina, 100 cycles kit, 20100987) on an Illumina NextSeq 1000 instrument. The quality of the sequencing run was assessed using Illumina Sequencing Analysis Viewer (Illumina version 2.4.7), and all base call files were demultiplexed and converted into FASTQ files using Illumina bcl2fastq conversion software v2.20. The quality control assessments, rRNA depletion, generation of libraries, sequencing, and demultiplexing were carried out at the Next Generation Sequencing Platform, University of Bern.

Raw RNA-Seq reads were first assessed for quality using FastQC v0.11^54^, followed by adaptor trimming with Trim Galore v0.6.11^55^. Quality was assessed post-trimming to ensure successful adapter removal and overall read integrity again using FastQC. Trimmed reads were aligned to the *S. pneumoniae* D39V reference genome (Accession: CP027540.1) using BWA-MEM v0.7, and sorted and indexed with SAMtools v1.15^56,57^. Gene-level counts were generated using FeatureCounts v1.6^58^. Normalization and differential expression analysis were performed using DESeq2 v1.34^59^. Differentially expressed genes between experimental conditions were defined as those with an adjusted *p*-value < 0.05 and an absolute log_2_ fold change > 2.

### Insertion of the pooled sgRNA library into *S. pneumoniae* D39 and R6

The used protocol can be found here^22^. In contrast to the IPTG-induced system, an aTc-inducible system was used in this study (see also ref. ^38^). The ready-made sgRNA pool (Addgene #170432) covers core operons of different pneumococcal strains, e.g., R6 has an estimated coverage of 90.3% of all annotated genetic elements^22^. In this study, the in-house D39 lab strain and R6 were used to generate a pooled CRISPRi library. The VL2212 (based on D39V) CRISPRi library has a frameshift in the *lacD* gene and is therefore unable to metabolize Gal^60^. Nonetheless, the VL2212 CRISPRi library was used to amplify DNA of *tetR* (inserted at the *prsA* locus, 2.3 kb, transformation and selection on gentamycin) as well as *dCas9* (inserted at the *bgaA* locus, 9.5 kb, transformation and selection on tetracycline). The primers used for strain construction in this study are listed in Table 2. The construction of all strains is detailed in Table 1. Constructs were checked by PCR, followed by gel electrophoresis and sequencing. To construct the pooled CRISPRi libraries, 200 ng of the sgRNA plasmid pool DNA was transformed into *S. pneumoniae* D39 and R6. The transformants were plated using sterile glass beads on 25 mL CSBA plates (containing 100 mg/mL spectinomycin), to have an average of >100 pneumococcal transformants per sgRNA. After overnight (14–16 h) incubation at 37 °C and 5% CO_2_, 6 plates of each transformant were harvested by scrapping them off the plates with fresh semi-defined C + Y medium, pH = 6.8^22^, (containing spectinomycin; 4 mL per plate), mixed with 80% glycerol (vol/vol) in a 4:1 (vol/vol) culture-to-glycerol ratio, and stored at −80 °C. Out of this stock, a preculture was created by adding 100 µL of the stock to 4 mL of fresh C + Y medium (with spectinomycin), incubating it in a water bath at 37 °C until an OD_600 nm_ of 0.3 was reached. This preculture was mixed with 80% glycerol (vol/vol) in a 4:1 (vol/vol) culture-to-glycerol ratio and stored at −80 °C.

### Generation of *lacG-2* deletion strain—construct

Serotype 2 (D39 and its non-encapsulated derivative, R6) and serotype 7F (208.41) strains were used to generate *lacG-2* deletion strains. Homologous arms were selected within the coding region of the *lacG-2* gene. The upstream (414 bp) and downstream homology arms (303 bp in D39 and in R6, and 304 bp in 208.41) were amplified by PCR using iProof™ High-Fidelity DNA Polymerase (#1725301—Bio-Rad). The used primers are presented in Table 2. Genomic DNA of the strains served as a template for amplification. For the initial denaturation, 98 °C was used for 3 min, followed by 35 cycles of 98 °C for 10 s, 55 °C for 30 s (annealing), and 72 °C for 30 s (extension). A final extension at 72 °C for 10 min was used. The kanamycin resistance cassette (920 bp) was amplified from the plasmid pGEM-T:Janus aphIII-rpsL+ using the primers DAM406 and DAM347^61^. All PCR products were verified by gel electrophoresis and purified using the QIAquick PCR Purification Kit (#28106—Qiagen). The upstream homology arm and the kanamycin resistance cassette were digested with BamHI-HF® (#R3136S—New England Biolabs), ligated using T4 DNA Ligase (#M0202L—New England Biolabs), amplified by PCR, and subsequently purified. The fused upstream-cassette product and the downstream homology arm were then digested with EcoRI (#R0101S—New England Biolabs), ligated using T4 DNA Ligase, amplified by PCR (yielding a final product of around 1600 bp for all strains), and purified. The production of the right construct was verified through Sanger sequencing (Microsynth, Switzerland).

### Transformation into *S. pneumoniae*

For transformation, the strains were cultivated in a 1:100 dilution in fresh C + Y medium at 37 °C and 5% CO_2_ until an OD_600 nm_ of 0.1. The competence of the cells was induced with the addition of 100 ng of CSP-1 (#AS-63779—Anaspec) and incubation at 37 °C for 12 min. Transformation was initiated by adding 100 ng of DNA construct to induced cells (cells without DNA were used as a negative control). Afterwards, cells were incubated for 20 min at 30 °C before adding 900 µL fresh C + Y and an additional incubation step for 1.5 h at 37 °C. Different dilutions were plated on CSBA plates supplemented with kanamycin and incubated overnight at 37 °C and 5% CO_2_. Colony PCR of single colonies was done using iProof™ High-Fidelity DNA Polymerase and respective primers (Table 2). PCR products with the correct length were purified and sent for Sanger sequencing (Microsynth, Switzerland). The construction of all strains is detailed in Table 1.

### Pooled CRISPRi fitness assay

A previously described protocol was used in this study^22^. However, not C + Y medium, but Lacks medium was used (Fig. 3A). A 1:100 dilution (preculture) of the aTc-inducible CRISPRi strains was used in 10 mL of media with either Glc or Gal as the sole C-source and grown at 37 °C to an OD_600 nm_ of 0.1. Next, a 1:10 dilution (induction) of the preculture was used to inoculate fresh 10 mL of media with either Glc or Gal (non-induced/control group). Additionally, another 10 mL of media is needed in which the inducer (aTc: #631310—TaKaRa) is added (induced/experiment group). As soon as an OD_600 nm_ of 0.1–0.2 was reached, the bacteria were centrifuged (3000 × *g* and 5 min) and washed twice with fresh media without C-source, before a 1:100 dilution was used to inoculate the treatment group. For the treatment group, different sugars ([25 mM]: Glc, Gal, Lac, 6′SL, 3′SL, and LNT; [100 mM]: 3-FL) were added as sole C-sources to the media. These two groups, the control and experiment groups, are each set up in quadruplicate. The bacteria were grown to mid-logarithmic phase before they were harvested by centrifugation (4122 × *g*, 15 min at 4 °C). The supernatant was removed, the pellet was resuspended in 1 mL of cold PBS, and transferred to an Eppendorf tube. Next, another centrifugation step (2400 × *g*, 5 min at 4 °C) with subsequent removal of the supernatant was conducted before the bacterial pellets were frozen at −80 °C for later gDNA isolation.

### Isolation of gDNA

For isolation of gDNA, a master mix had to be prepared with the following solutions (for one sample): 800 µL Nuclei Lysis Solution (#A7943—Promega), 4 µL SDS [10%], 2 µL DOC [10%], and 16 µL RNase A (#EN0531—ThermoFisher). To each sample, 822 µL of the master mix was added, and incubated for 20 min at 37 °C. Next, additional incubation steps for 5 min at 80 °C followed by 10 min at 37 °C were performed. Lastly, the sample had to be cooled down to room temperature. 250 µL Protein Precipitation Solution (#A7953—Promega) was added, and the sample was vortexed vigorously at high speed for 20 s before being incubated on ice for 10 min. After centrifugation at 14,000 × *g* for 10 min at 4 °C, the supernatant containing the DNA was transferred to a clean 2 mL Eppendorf tube containing 600 µL isopropanol (#1.09634.2500—Merck). After gentle inversion of the tube, which led to the formation of thread-like strands of DNA, an additional centrifugation step was conducted before the supernatant was discarded, and 600 µL ethanol [70%] was added to the DNA pellet for washing. Another centrifugation step was performed (only 5 min) before the supernatant was again discarded, and the DNA pellet was allowed to air-dry for at least 15 min. 100 µL RNase- and DNase-free water was used to rehydrate the DNA by incubating at 65 °C for 15 min. gDNA concentration was measured using a NanoDrop One^C^ spectrophotometer (ThermoScientific) before the gDNA was stored at −20 °C.

### Library preparation for next-generation sequencing

Illumina libraries were prepared using a one-step PCR with gDNA as template (used Primers in ref. ^22^—Table 1). 2 µL of primers and 10 µL of gDNA were used for each 50 µL PCR reaction with 25 µL 2× Phanta Master Mix (#P515-02—Vazyme). After a denaturation step at 95 °C for 1 min, a 30× loop consisting of 15 s at 95 °C, 15 s at 55 °C (annealing), and 1 min at 72 °C (elongation), followed by 10 min at 72 °C was conducted. The PCR amplicons (303 bp) were purified from a 2% agarose gel using Macherey-Nagel NucleoSpin Gel and PCR Clean-up Kit (#REF 740609.50—Macherey-Nagel). The amount of DNA was quantified by Qubit assay (Q33231—Invitrogen). The purified libraries were then pooled together, and the molar concentration of the purified amplicons was calculated by converting the concentration in ng/µL to nM using the following formula (1):

$$\frac{({Concentration\; in}\frac{{ng}}{{\rm\mu }L})}{(660\frac{g}{{mol}}{x\; Amplicon\; size\; in\; bp}\left({here}303{bp}\right))}x{10}^{6}={Concentration\; in\; nM}$$

The library was then diluted with a resuspension buffer (10 mM Base Trizma® (#T1503—Merck), pH 8.5 with HCl, and a final 0.1% Tween® 20 (#P1379—Merck) concentration) to a final pooled library concentration of 10 nM.

The quality control assessments, rRNA depletion, generation of libraries, and sequencing were carried out at the Next Generation Sequencing (NGS) platform (University of Bern).

At the NGS platform, the library pool was evaluated using a Thermo Fisher Scientific Qubit 4.0 fluorometer with the Qubit dsDNA HS Assay Kit (Thermo Fisher Scientific, Q32854) and an Agilent Fragment Analyzer (Agilent) with an HS NGS Fragment Kit (Agilent, DNF-474), respectively.

The libraries were sequenced using various shared Illumina NovaSeq 6000 S1 Reagent Kit v1.5 (100 cycles; Illumina, 20028319) on an Illumina NovaSeq 6000 instrument. The read set-up was 114/12/12/0 or 113/12/12/101, and the libraries were run alongside many other, more complex NGS libraries. Some libraries were also sequenced alone on a NextSeq 1000/2000 P1 Reagent (100 cycles) (Illumina, 20074933) using an Illumina NextSeq 1000 instrument. The run had a read set up of 122/8/8/0, and the first 54 cycles were run as dark cycles according to a custom recipe. The quality of the sequencing run was assessed using the Illumina Sequencing Analysis Viewer (version 2.4.7). All base call files were demultiplexed and converted into FASTQ files using Illumina bcl2fastq conversion software v2.20.

### Data analysis of CRISPRi sequencing

For sgRNA counting, the 2FAST2Q tool (v2.5.5) with the following settings: minimum Phred score 25, one mismatch allowed, was used^62^. The sgRNAs were annotated using the curated file previously described^22^. The differential fitness analysis was performed with the DESeq2 package (v1.48.1)^59^ in R (v4.5.0). The used absolute log2FC cut-off was set at 1 with a *p* < 0.05. Three replicates with fewer than 100,000 reads were excluded from the analysis, and the data for the R6 strain resulted from two batches. However, replicates are compared to uninduced ones separately for every condition. Therefore, each condition has its own “baseline” sgRNA abundance defined by the uninduced replicates for that condition from the same batch.

### NMR spectra for the measurements of metabolites

For the extraction of extracellular metabolites (^1^H-NMR), pneumococcal growth (strain 106.66 (6B) in CDM) of three different growth phases (early-, mid-, and late-log phase) in LNnT, Lac and 3-FL were collected and centrifuged at 3000 × *g* for 5 min at room temperature. The supernatant was sterile filtered through a Merck Millipore Millex GV syringe filter, dried under reduced pressure overnight, and dissolved in 0.5 mL D_2_O containing 2 mM Trimethylsilylpropanoic acid (TSP) and transferred into 1.7 or 5 mm NMR tubes. NMR data were collected on a Bruker Avance II (500 MHz; ^1^H) spectrometer equipped with a 1.7 mm triple-resonance (^1^H, ^13^C, ^31^P) microprobe head or on a Bruker AvanceNEO (400 MHz; ^1^H) spectrometer equipped with a triple resonance broadband observe (TBO, ^1^H, X, ^19^F) 5 mm probe head. 500 MHz data (Figs. S13 and S14): ^1^H spectra were acquired using the pulse sequence *zgpr*, using 1024 scans with a spectral width of 12500.0 Hz, a recycling delay of 1 s, and an acquisition time of 1.311 s. All spectra were acquired at a regulated temperature of 298 K. Residual water suppression was achieved using a classical presaturation procedure, by applying a weak, selective radiofrequency pulse at the water’s resonance frequency during the relaxation delay. Experiments were recorded using the TopSpin® software, version 3.2 (Bruker Biospin), and processed using TopSpin® version 4.0.7. 400 MHz data (Fig. 4): ^1^H spectra were acquired using the pulse sequence *noesygppr1d* with mixing times of 20 ms, using 1024 scans with a spectral width of 8196.79 Hz, a recycling delay of 8 s, and an acquisition time of 1.998 s. All spectra were acquired at a regulated temperature of 298 K. Residual water suppression was achieved using a classical presaturation procedure, by applying a weak, selective radiofrequency pulse at the water’s resonance frequency during the relaxation delay. All experiments were recorded using the TopSpin® software, version 4.1 (Bruker Biospin), and processed using TopSpin® version 4.4.

Quantification was performed using TSP (2 mM) as an internal standard. Analyte concentrations were calculated from the ratio of the analyte and TSP integrals, taking into account the number of protons contributing to each signal (9 for TSP) and applying a correction factor of 3 to account for sample concentration prior to NMR analysis. Free α-galactose was quantified using the anomeric proton signal at 5.2 ppm as described^63^. Residual LNnT was monitored using the β-galactose H1 signal at approximately 4.5 ppm, and formate was quantified using its proton resonance at 8.4 ppm.

### Quantification of H_2_O_2_ production

In order to further evaluate the metabolism of HMOs by the pneumococcus, D39 was grown in Lacks and 25 mM of C-sources (Gal, Glc, Lac, 6′SL, 3′SL, and LNnT). As soon as the mid-log phase (OD_600 nm_ of 0.4) was reached, the cells were pelleted at 3000 × *g* for 10 min at 4 °C and washed twice with Hanks balanced medium (pH 7.4). The Amplex Red hydrogen peroxide/peroxidase assay kit (Molecular Probes, Eugene, OR) was used to measure the H_2_O_2_ concentration in the supernatant as described before^64^.

### Epithelial adherence assay with Detroit 562 cells

Bacterial cells (R6) were harvested at mid-log phase in Lacks medium and Gal [20 mM]. Bacteria were centrifuged and washed once with PBS, then resuspended in Dulbecco’s MEM (Thermo Fisher Scientific, USA). Suspension in MEM was diluted to reach the target OD_600 nm_ with a bacterial inoculum of 1.8 × 10^5^ bacteria/mL. Subsequently, 500 μL of this inoculum and 500 μL MEM ( ± Lac or HMOs) were added to the wells of a 24-well plate containing Detroit 562 cells (seeding density: 3 × 10^5^ cells per well) from ATCC (American Type Culture Collection, USA). The 24-well plates with bacteria and Detroit cells were incubated for 1 h at 37 °C and 5% CO_2_. Then, the medium was removed, and the wells were washed 5 times with PBS. Cells were detached by adding 200 μL of trypsin-EDTA (0.05%, Thermo Fisher Scientific, USA) to each well and incubating for 5 min at 37 °C. MEM (800 μL) was added, and serial dilutions of bacteria per cell suspension were done. 10 μL of the dilutions was then streaked on CSBA plates and incubated overnight. Adherence of bacteria was calculated the following day. The data includes 3 measurements (technical triplicates) of 3 biological replicates (9 data points in total) for each experiment. Tests used to determine statistical significance for each data set are indicated in the respective figure captions. Statistical analyses were performed using GraphPad Prism 10.4.1 software

### Epithelial adherence assay with primary nasal epithelial cells cultured at the air-liquid interface

Primary nasal epithelial cells of adult healthy volunteers living in the area of Bern, Switzerland were cultured using PneumaCult media (Stemcell technologies) as previously described in ref. ^65^. All procedures were performed in compliance with relevant laws and institutional guidelines and have been approved by the ethics committee of the Canton Bern (Kantonale Ethikkommission Bern), Switzerland (project identification code 2018-02155). We obtained written information consents from all participants. Cells were cultured for at least 28 days at the air-liquid interface before being subjected to the adherence assay. After the performance of the assays, the cells were destroyed.

Bacterial cells (R6) were harvested at mid-log phase in Lacks medium and Gal [20 mM]. Bacteria were centrifuged and washed twice with Dulbecco’s phosphate-buffered saline (DPBS; Thermo Fisher Scientific, USA). 40 μL of bacterial suspension in DPBS (± 2′FL in different concentrations) of approximately 10^6^ bacterial cells was added per insert. Before the bacteria were added, the primary nasal epithelial cells were carefully washed three times with 200 μL DPBS to get rid of the mucus. After 24 h, the inserts were again washed three times with DPBS to remove all non-adhering bacteria. Cells were detached by adding 100 μL of trypsin-EDTA to the apical side and incubating for 10 min at 37 °C. DPBS (400 μL) was added, and serial dilutions of bacteria per cell suspension were made. 10 μL (3×) of the dilutions was then streaked on CSBA plates and incubated overnight at 37 °C and 5% CO_2_. Adherence of bacteria was calculated the following day. Statistical analyses were performed using GraphPad Prism 10.4.1 software.

### Mouse colonization experiments

Animal experiments were executed at the University of Dundee, with prior approval of the UK Home Office and the Animal Welfare and Ethical Review Board. Experiments were carried out under project licenses PP2072053 and PP4318217. The principles of the Declaration of Helsinki were observed throughout. Environmental enrichment was provided in all cages, and mice were acclimatized to the animal unit for at least 10 days before use. The animals were housed under the following conditions; temperature was 21–23 °C and humidity set at 55–65%, 12 h light-dark cycle. Mice were placed in individually ventilated cages, provided with access to pelleted food and fresh sterile water, ad libitum and enrichment was included in all cages, including nesting material, a domed home and handling tunnel. Mice were randomly allocated to cages on arrival in the animal unit by staff with no role in study design.

CD1 (6 to 7-week-old) female mice were intranasally infected under light anaesthesia (oxygen and isoflurane) with 1 × 10^5^ colony-forming units (CFU) in 10 μL PBS (nasopharyngeal colonisation model). In some experiments, infections were performed in saline containing 200 mM Lac or 2-FL and further intra-nasal administrations of HOs in 10 µl saline were given at days 4 and 10 post-infection. Disease severity was regularly monitored using a scoring system evaluating appearance, behaviour, breathing, and weight loss. Mice were euthanized via CO₂ and cervical dislocation either at pre-determined time points or when they reached severity endpoints. Blood was collected by cardiac puncture under terminal anaesthesia, and nasopharynx and lungs were harvested in PBS for CFU determination. For the determination of CFU in blood and organs, ten-fold serially diluted tissue samples were plated onto blood agar plates (Oxoid) containing 5% defibrinated horse blood and 40 μg/mL gentamycin (Sigma). Plates were incubated overnight at 37 °C in an anaerobic jar, and bacterial colonies were counted the next day. For experiments reported in this manuscript, individual mice were considered as the experimental unit. Statistical analyses were performed using GraphPad Prism 10.4.1 software.

### Reporting summary

Further information on research design is available in the Nature Portfolio Reporting Summary linked to this article.

## Supplementary information

Supplementary Figs.

Description of Additional Supplementary Files

Dataset 1

Dataset 2

Dataset 3

Dataset 4

Dataset 5

Dataset 6

Dataset 7

Dataset 8

Reporting Summary

Transparent Peer Review file

## Source data

Source data

## Data Availability

Raw CRISPRi-seq data is available on SRA, accession number PRJNA1337430. The raw RNA sequencing data can be downloaded from SRA, accession number PRJNA1452587. Source data are provided with this paper.

## Code availability

All code and scripts for analysis and figures are available as a code repository on GitHub (https://github.com/jburgaya/2026_transcriptomics_HMOs.git).
