# Supplementary material for: Metabolization of human milk oligosaccharides by Streptococcus pneumoniae
Source: Nat Commun. 2026 Sep 30;17:11242. doi: 10.1038/s41467-026-77970-7 (PMC13720028; doi:10.1038/s41467-026-77970-7)
Supplement: Supplementary file 1 [file 41467_2026_77970_MOESM11_ESM.pdf]

nature portfolio

Corresponding author(s): Markus Hilty

Last updated by author(s): Aug 10, 2026

Reporting Summary

Nature Portfolio wishes to improve the reproducibility of the work that we publish. This form provides structure for consistency and transparency in reporting. For further information on Nature Portfolio policies, see our Editorial Policies and the Editorial Policy Checklist.

Statistics

For all statistical analyses, confirm that the following items are present in the figure legend, table legend, main text, or Methods section.

n/a	Confirmed	
☐	☒	The exact sample size (n) for each experimental group/condition, given as a discrete number and unit of measurement
☐	☒	A statement on whether measurements were taken from distinct samples or whether the same sample was measured repeatedly
☐	☒	The statistical test(s) used AND whether they are one- or two-sided<br>*Only common tests should be described solely by name; describe more complex techniques in the Methods section.*
☐	☒	A description of all covariates tested
☐	☒	A description of any assumptions or corrections, such as tests of normality and adjustment for multiple comparisons
☐	☒	A full description of the statistical parameters including central tendency (e.g. means) or other basic estimates (e.g. regression coefficient) AND variation (e.g. standard deviation) or associated estimates of uncertainty (e.g. confidence intervals)
☐	☒	For null hypothesis testing, the test statistic (e.g. F, t, r) with confidence intervals, effect sizes, degrees of freedom and P value noted<br>*Give P values as exact values whenever suitable.*
☒	☐	For Bayesian analysis, information on the choice of priors and Markov chain Monte Carlo settings
☒	☐	For hierarchical and complex designs, identification of the appropriate level for tests and full reporting of outcomes
☐	☒	Estimates of effect sizes (e.g. Cohen's d, Pearson's r), indicating how they were calculated

Our web collection on statistics for biologists contains articles on many of the points above.

Software and code

Policy information about availability of computer code

Data collection	Software used: Illumina bcl2fastq Conversion Software v2.20
Data analysis	Software used: FastQC v0.11, 2FAST2Q v2.5.2, Trim Galore v0.6.11, BWA-MEM v0.7, SAMtools v1.15, FeatureCounts v1.6, R package DESeq2 v1.34 or v1.48.1, R v4.5.0. All code and scripts for analysis and figures are available as a code repository on GitHub (https://github.com/jburgaya/2026_transcriptomics_HMOs.git).

For manuscripts utilizing custom algorithms or software that are central to the research but not yet described in published literature, software must be made available to editors and reviewers. We strongly encourage code deposition in a community repository (e.g. GitHub). See the Nature Portfolio guidelines for submitting code & software for further information.

Data

Policy information about availability of data

All manuscripts must include a data availability statement. This statement should provide the following information, where applicable:

- Accession codes, unique identifiers, or web links for publicly available datasets
- A description of any restrictions on data availability
- For clinical datasets or third party data, please ensure that the statement adheres to our policy

Raw CRISPRi-seq data is available on SRA, accession number PRJNA1337430. The raw RNA sequencing data can be downloaded from SRA, accession number PRJNA1452587. Source data are provided with this paper.

Research involving human participants, their data, or biological material

Policy information about studies with human participants or human data. See also policy information about sex, gender (identity/presentation), and sexual orientation and race, ethnicity and racism.

Reporting on sex and gender	Generally, we did not perform research involving human participants, their data, or biological material. However, we received primary nasal epithelial cells of three adult healthy volunteers. The sex was kept anonymous.
Reporting on race, ethnicity, or other socially relevant groupings	N/A
Population characteristics	Primary nasal epithelial cells of three adult healthy volunteers were received.
Recruitment	Participants were randomly selected. We obtained written information consents from all the participants. After the performance of the assays, the cells were destroyed.
Ethics oversight	All procedures were performed in compliance with relevant laws and institutional guidelines and have been approved by the ethics committee of the Canton Bern (Kantonale Ethikkommission Bern), Switzerland (project identification code 2018-02155). We obtained written information consents from all participants.

Note that full information on the approval of the study protocol must also be provided in the manuscript.

Field-specific reporting

Please select the one below that is the best fit for your research. If you are not sure, read the appropriate sections before making your selection.

[x] Life sciences [ ] Behavioural & social sciences [ ] Ecological, evolutionary & environmental sciences

For a reference copy of the document with all sections, see nature.com/documents/nr-reporting-summary-flat.pdf

Life sciences study design

All studies must disclose on these points even when the disclosure is negative.

Sample size	A minimum of three biological replicates was used for all experiments, which was sufficient to observe reproducible results and is indeed a standard in the field, culminating from extensive research demonstrating reproducibility for the types of assays performed in this study, e.g., bacterial growth assays.
Data exclusions	No data were excluded from analysis.
Replication	All experiments were performed with at least three biological replicates, on top of varying numbers of technical replicates depending on assay type. All data is shown in figures to confirm reproducibility.
Randomization	No blinding was applied as it was not necessary or practical, and no obvious covariates needed to be accounted for given the assay types used in this work, e.g., bacterial growth assays.
Blinding	No blinding was applied as it was not necessary or practical, and there were no obvious strong biases that could specifically be prevented by blinding given the assay types used in this work, e.g., bacterial growth assays.

Reporting for specific materials, systems and methods

We require information from authors about some types of materials, experimental systems and methods used in many studies. Here, indicate whether each material, system or method listed is relevant to your study. If you are not sure if a list item applies to your research, read the appropriate section before selecting a response.

Materials & experimental systems

n/a	Involved in the study	
[x]	[ ]	Antibodies
[ ]	[x]	Eukaryotic cell lines
[x]	[ ]	Palaeontology and archaeology
[ ]	[x]	Animals and other organisms
[x]	[ ]	Clinical data
[x]	[ ]	Dual use research of concern
[x]	[ ]	Plants

Methods

n/a	Involved in the study	
[x]	[ ]	ChIP-seq
[x]	[ ]	Flow cytometry
[x]	[ ]	MRI-based neuroimaging

Eukaryotic cell lines

Policy information about cell lines and Sex and Gender in Research

Cell line source(s)	Detroit 562 Cell Line
Authentication	STR Authentication
Mycoplasma contamination	The cells were not tested for mycoplasma contamination
Commonly misidentified lines (See ICLAC register)	*Name any commonly misidentified cell lines used in the study and provide a rationale for their use.*

Animals and other research organisms

Policy information about studies involving animals; ARRIVE guidelines recommended for reporting animal research, and Sex and Gender in Research

Laboratory animals	CD1 female mice (4–6 weeks old, Charles River, UK)
Wild animals	The study did not involve wild animals.
Reporting on sex	Sex based analyses were not performed as we investigated carriage only.
Field-collected samples	No samples collected from the field.
Ethics oversight	Animal experiments were executed at the University of Dundee, with prior approval of the UK Home Office and the Animal Welfare and Ethical Review Board. Experiments were carried out under project licenses PP2072053 and PP4318217. The principles of the Declaration of Helsinki were observed throughout. Mice were housed in individually ventilated cages, with access to food and water ad libitum. Environmental enrichment was provided in all cages, and mice were acclimatized to the animal unit for at least 10 days before use. Mice were randomly allocated to cages on arrival in the animal unit by staff with no role in study design.

Note that full information on the approval of the study protocol must also be provided in the manuscript.

Plants

Seed stocks	N/A
Novel plant genotypes	N/A
Authentication	N/A
