# Supplementary material for: Metabolization of human milk oligosaccharides by Streptococcus pneumoniae
Source: Nat Commun. 2026 Sep 30;17:11242. doi: 10.1038/s41467-026-77970-7 (PMC13720028; doi:10.1038/s41467-026-77970-7)
Supplement: Supplementary file 2 [file 41467_2026_77970_MOESM12_ESM.pdf]

Peer Review File

Metabolization of human milk oligosaccharides by Streptococcus pneumoniae

Corresponding Author: Dr Markus Hilty

This file contains all reviewer reports in order by version, followed by all author rebuttals in order by version.

Version 0:

Reviewer comments:

Reviewer #1

(Remarks to the Author)
The important human pathogen Streptococcus pneumoniae relies on host glycoconjugates for its survival. One of the most notable glycoconjugates that S. pneumoniae encounter include dietary human milk oligosaccharides (HMO). The authors investigated how these HMOs are metabolised, their role in capsule biosynthesis and whether HMO can be used to prevent adherence. They found that the carbon catabolite repression system is important for HMO metabolism. A genome-wide CRISPRi screen revealed the lacEF-2 operon is essential for metabolizing all HMOs except 2'-Fucosyllactose (2FL). Using 13C-labelled lactose, they traced glucose and galactose incorporation into the capsule. Gene knockouts in lacEF-2 did not affect adherence or virulence in vivo. Notably, 2FL inhibited adherence to epithelial cells, suggesting its potential as a prebiotic to prevent pneumococcal infection.

This is a good article and will contribute to the field. However, there are certain minor points that the authors need to improve to enhance the clarity as listed below:

Major and minor points:

1. Ln. 79: Give some background information about for CRISPRi-seq. How it works and why it is promising.
2. Ln 84. Indicate who has low abundance of Bifidobacterium
3. Ln. 117-118. Discuss this statement as to why growth with these two HMO is 'reduced' even at high concentration: 'However, growth with LNT and, especially, 3FL, even at a higher concentration (100mM), is reduced compared to the other HMOs (25 mM) (Fig. 1, C).
4. Ln.155: In figure 1, there is no D39 data. Please clarify.
5. Ln158-162: Briefly discuss why exoglycosidases have different affinity for different substrates.
6. Ln 168: Correct: Acetylneuraminidase (NeuNAc) as Acetylneuraminic acid (NeuNAc).
7. Ln. 181-182: Define what is moderate growth or good growth. Give the growth yield instead.
8. Ln. 289: Justify why R6 strain was used.
9. Ln. 307: Give the p value
10. Ln. 349: Discuss why CRISPR screen did not identify the essential genes for 3FL.

Reviewer #2

(Remarks to the Author)
The manuscript is based on the hypothesis that HMO may be present in the upper airway of breastfed infants and that these carbohydrates could influence the development of pathogens such as S. pneumoniae. However, these assumptions remain to be validated. The manuscript addresses multiple topics (such as the CCR of HMO utilization, the identification of genes involved in HMO catabolism, the incorporation of glucose and galactose from lactose into the capsule, and the impact of HMO utilization on infectivity and adhesion) which makes it appear somewhat unfocused. Moreover, it does not provide conclusive results or mechanistic explanations for most of these aspects.
Specific points
Instead of testing CCR by using cells pre-grown on glucose or galactose before exposure to HMOs, a more informative approach would have been to grow the strain in the presence of two sugars simultaneously and monitor diauxic growth,

sequential utilization of carbon sources, or changes in gene expression. While poor or absent growth after pre-growth on glucose may suggest CCR, it is not a definitive evidence. It would also be relevant to examine whether genes involved in HMO metabolism contain cre sites in their promoters. Additionally, testing a ccpA mutant strain could have provided stronger support for CCR involvement.
This reviewer acknowledges the effort invested in applying CRISPRi technology to identify genes required for HMO utilization. However, one might question whether a more classical approach: starting with a genome-wide search for glycoside hydrolases and transporters likely involved in HMO uptake and hydrolysis, followed by targeted mutagenesis, would have been more straightforward and likely to reveal key players such as the lacEFG genes. Moreover, the CRISPRi strategy did not succeed in identifying potential transporters for 3FL and LNT, which remains a significant gap.
HMO utilization was examined, and it was shown that 2FL,despite not being metabolized by S. pneumoniae, inhibits the pathogen cellular adhesion. However, no mechanistic explanation for this observation is provided. Several HMO are known to act as decoys, preventing pathogen binding to host cells, but in the case of S. pneumoniae, the specific adhesin(s) likely targeted by 2FL or lactose have not been identified or experimentally confirmed. It was hypothesized that non metabolizable HMO, such as 2FL would show increased inhibition of bacterial adhesion to host cells, compared to metabolizable sugars such as lactose. However, no differences in inhibition were seen for 2FL and lactose in Detroit 562 cells. Why lactose was not tested in the ALI cell culture model?
The manuscript mentions eIIA and eIIC genes, but their identity and functional roles are not adequately explained. Additionally, the infection experiments using the lacG mutant should have included a lacE mutant as a positive control, since previous studies have reported that lacE deletion reduces the ability to infect mice. It is also unclear which components of the PTS are encoded by lacE and lacF; this should be specified. Furthermore, lacE and lacF (?) mutants were used in cell adhesion assays, yet no differences were observed compared to the wild type. The rationale for expecting a lactose transporter to influence adherence is not evident.

Reviewer #3

(Remarks to the Author)
In this manuscript by Bacher et al. the investigators identify and then examine the role of the PTS lactose operon during growth of S. pneumoniae in the presence of human milk oligosaccharides. This is relevant as infants, are presumably exposed to these oligosaccharides, during nursing impacting the bacterium’s physiology. The methodology for included experiments is robust, however, the authors make claims that are not robustly supported by the included results. These are detailed below. Completion of the additional experiments needed to support their claims would vastly improve the manuscript.
1) The authors claim that growth on HMO requires carbon catabolite derepression. This is supported by the observation that pre-growth of the bacteria in glucose containing media impairs Spn outgrowth in HMO-supplemented media, whereas pre growth in galactose containing media favors growth. This is circumstantial and it does not directly show that CCR must be derepressed during growth in the presence of HMO. For example, authors could measure H_2O_2 which is produced in greater amounts during mixed fermentation, comparing these to glucose and galactose controls. qRT-PCR for lacO, pfl and spxB would also be a way to show this.
2) Along such lines, the authors should show that the lac operon is also expressed at higher levels during growth conditions associated with CCR derepression.
3) Its also unclear if on line 241 if the authors mean that 81-92% of all the labeled carbon became associated with polysaccharide. Or if instead 81-92% of de novo capsule was made of labeled sugars. Does polysaccharide mean just capsule or capsule, teichoic acid, peptidoglycan, all of which are polysaccharides?
4) Give than lac metaboization is dispensable during in vivo experimentation it is suprising that the authors did not reexamine their postulated model that HMOs may find their way to the respiratory tract and this may be a boon for bacteria that can degrade these carbon sources. Without this finding the rationale proposed for the paper and title seems to be unmerited. Perhaps examining colonization levels in mice that are instilled intranasally with HMOs is a way to show that the WT can outgrow the lac operon mutant under these conditions? Alternatively use a nursing pup model of colonization to test this.
5) The adhesion experiment to cell lines were done using R6 and not D39. R6 is 100-fold more adhesive than D39 and this may obfuscate important results.
6) The authors show that 2FL blocks adherence in vitro, but not necessarily how. These experiments should be repeated using the lac operon deficient mutant so that at least the transporter itself can be ruled in/out.
7) Again, and given the lack of in vivo validation, the authors should consider treating colonized mice with 2FL and determining if this impacts colonization burden.

Version 1:

Reviewer comments:

Reviewer #2

(Remarks to the Author)
The revised manuscript has been substantially improved, including additional experiments and removing data that somewhat detracted from the main focus (e.g., sugar incorporation into the capsule). My primary concern in the original version was the lack of mechanistic insights. While the possibility that HMOs are present in the upper airways of breastfed infants remains speculative and is not directly demonstrated, the authors now provide new in vivo evidence in mice showing

that lactose and 2FL affect the persistence of S. pneumoniae strains. In this regard, it should be noted that lactose is not an HMO, and this should be corrected in Figure 6.
Concerning CCR during HMO utilization, the authors have added new growth curves and RNA-seq analyses of gene expression, providing a more comprehensive understanding of HMO metabolism in S. pneumoniae. In addition, several inaccuracies and insufficiently explained points from the previous version have been addressed.
Despite these improvements, I still believe that the most significant finding of the study, namely that HMOs may inhibit S. pneumoniae colonization, remains insufficiently explored at the mechanistic level. The work is technically sound and carefully executed, but it largely remains descriptive. Specifically, the manuscript identifies genes involved in HMO utilization through CRISPRi-seq, characterizes their transcriptional regulation in response to different HMO, examines CCR in the presence of glucose (growth curves), and demonstrates that the non-metabolizable HMO (2FL) reduces bacterial adhesion in vitro and pneumococcal carriage in a mouse model. However, the molecular basis of this inhibitory effect is not investigated. It remains unclear which pneumococcal adhesins or other bacterial factor(s) may mediate the observed reduction in adhesion and colonization. Likewise, the potential impact of metabolizable HMOs on colonization (inhibitory or stimulatory) is not addressed.
The manuscript covers two distinct topics: the genetic basis of HMO utilization and the effect of HMO on S. pneumoniae adhesion and colonization. However, neither aspect provides sufficiently conclusive or mechanistic insights.
For example:
The observation that the PTS transporter encoded by lacEF is not essential for LNT utilization is not adequately explained. This transporter is required for the utilization of other HMOs such as LNnT, 3SL and 6SL. According to the model presented in Figure 7, all tested HMO are processed by extracellular glycosidases, releasing lactose, which is then imported through LacEF. If LNT metabolism also generates lactose, why does it differ from the other HMO in its dependence on LacEF? This apparent inconsistency requires clarification.
Could certain HMO serve as readily available carbon sources that enhance bacterial growth and persistence in vivo? The animal experiments focus exclusively on the inhibitory HMO 2FL. However, several HMO are efficiently metabolized by S. pneumoniae. Testing whether these compounds promote growth, colonization, or persistence in the mouse model would be highly informative and would provide a more balanced assessment of the biological consequences of HMO utilization.

Reviewer #3

(Remarks to the Author)
The authors have addressed my concerns. This is a very nice manuscript.

Open Access This Peer Review File is licensed under a Creative Commons Attribution 4.0 International License, which permits use, sharing, adaptation, distribution and reproduction in any medium or format, as long as you give appropriate credit to the original author(s) and the source, provide a link to the Creative Commons license, and indicate if changes were made.
In cases where reviewers are anonymous, credit should be given to 'Anonymous Referee' and the source.
The images or other third party material in this Peer Review File are included in the article's Creative Commons license, unless indicated otherwise in a credit line to the material. If material is not included in the article's Creative Commons

license and your intended use is not permitted by statutory regulation or exceeds the permitted use, you will need to obtain permission directly from the copyright holder.
To view a copy of this license, visit https://creativecommons.org/licenses/by/4.0/

<u>Point-by-point response to the reviewers' comments</u>

<u>Reviewer #1 (Remarks to the Author):</u>

The important human pathogen Streptococcus pneumoniae relies on host glycoconjugates for its survival. One of the most notable glycoconjugates that S. pneumoniae encounter include dietary human milk oligosaccharides (HMO). The authors investigated how these HMOs are metabolised, their role in capsule biosynthesis and whether HMO can be used to prevent adherence. They found that the carbon catabolite repression system is important for HMO metabolism. A genome-wide CRISPRi screen revealed the lacEF-2 operon is essential for metabolizing all HMOs except 2'-Fucosyllactose (2FL). Using 13C-labelled lactose, they traced glucose and galactose incorporation into the capsule. Gene knockouts in lacEF-2 did not affect adherence or virulence in vivo. Notably, 2FL inhibited adherence to epithelial cells, suggesting its potential as a prebiotic to prevent pneumococcal infection.

This is a good article and will contribute to the field. However, there are certain minor points that the authors need to improve to enhance the clarity as listed below:

Answer: Thank you for the encouraging feedback. Please note, we have added a lot of new data taking into account the suggestions of all reviewers.

Major and minor points:

1. Ln. 79: Give some background information about for CRISPRi-seq. How it works and why it is promising.

Answer: Yes, we have added some more information. CRISPRi-seq is a promising, unbiased genome-wide method for investigating gene fitness through different conditions. The strong advantage of this approach is the inducibility of the knockdown expression system that permits a resolution even on essential genes (in contrast to Tn-seq). (lines 77-80)

2. Ln 84. Indicate who has low abundance of Bifidobacterium

Answer: Sorry for the confusion. Bifidobacterium is at low abundance in adults. We deleted this information to not confuse the reader.

3. Ln. 117-118. Discuss this statement as to why growth with these two HMO is 'reduced' even at high concentration: 'However, growth with LNT and, especially, 3FL, even at a higher concentration (100mM), is reduced compared to the other HMOs (25 mM) (Fig. 1, C).

Answer: We meant to say that even if the concentrations are increased to 100mM, growth with LNT and 3FL is still lower as compared to the other HMOs. We made this clearer in the text. (lines 145-149)

4. Ln.155: In figure 1, there is no D39 data. Please clarify.

Answer: This is correct and has been changed as R6 and D39 data are newly illustrated in the supplementary figures 1C and 1D.

5. Ln158-162: Briefly discuss why exoglycosidases have different affinity for different substrates.

Answer: Exoglycosidases have different affinities for different substrates primarily due to the specific, complex structure of their active sites, which are designed to recognize unique glycan (sugar) types and the specific anomeric linkages at the non-reducing ends of carbohydrate chains [1]. This has now been better clarified in the revised manuscript. (lines 61-65)

6. Ln 168: Correct: Acetylneuraminidase (NeuNAc) as Acetylneuraminic acid (NeuNAc).

Answer: Well spotted. Corrected. Actually, we now try to consistently use 'Sia' for NeuNAc.

7. Ln. 181-182: Define what is moderate growth or good growth. Give the growth yield instead.

Answer: Moderate and very good growth means that the OD_{max} has been around 0.2 (for 3FL and LNT) and 0.8 (OD_{max} for Lac, 3SL, and 6SL), respectively. We added this important information to the text. (lines 235-238)

8. Ln. 289: Justify why R6 strain was used.

Answer: We used R6 as the strain is non-encapsulated (like the 415, 435 and 382 strains). However, we have deleted this particular data from the manuscript (which was mentioned in line 289ff). In contrast, new adherence data has been added to underline the importance of inhibition.

9. Ln. 307: Give the p value

Answer: Thank you. We added the p values in this section.

10. Ln. 349: Discuss why CRISPR screen did not identify the essential genes for 3FL.

Answer: Please note, we added the following (lines 468-473): This situation might be explained by transporter redundancy. In this case, knocking down only one transporter gene with a single sgRNA would not affect growth/fitness. Future work, for instance applying dual CRISPRi-seq [2], may provide insights into transporter redundancy. However, considering that our ^{1}H-NMR data indicates that 3FL is

not consumed, we hypothesize that the observed minor growth in the presences of high concentrations could also be due to 'contaminating' metabolites rather than actual 3FL

Reviewer #2 (Remarks to the Author):

The manuscript is based on the hypothesis that HMO may be present in the upper airway of breastfed infants and that these carbohydrates could influence the development of pathogens such as S. pneumoniae. However, these assumptions remain to be validated. The manuscript addresses multiple topics (such as the CCR of HMO utilization, the identification of genes involved in HMO catabolism, the incorporation of glucose and galactose from lactose into the capsule, and the impact of HMO utilization on infectivity and adhesion) which makes it appear somewhat unfocused. Moreover, it does not provide conclusive results or mechanistic explanations for most of these aspects. **Answer: Many thanks for the feedback. We agree that important facts were missing and appreciate the opportunity for a thorough point-to-point reply. First, we agree that we originally did not show data that HMOs have a direct effect on metabolization and/or adherence *in vivo.* Prior investigations have shown that HMOs are partly disseminated and can be found in the blood upon breastfeeding. Blood contains high amounts of glucose and, therefore, adding growth data including glucose and single HMOs are crucial. Also, we hypothesize that HMOs can be found in the mucus and in the nasopharynx, the natural niche for *S. pneumoniae*. Therefore, we think that the investigation on the role of HMOs for metabolization and adherence is both, very important and underexplored. We added more information to the revised manuscript (added in lines 83-90).**

Importantly, we have newly added some *in vivo* data from mice which suggest that non- or slowly metabolizable HMOs (e.g., 2FL) could provide increased adherence inhibition compared to metabolizable HMOs (e.g., Lac) (revised figure 6).

As for the issue addressing multiple topics and to make the manuscript more focused, we have newly excluded the section regarding the incorporation of glucose and galactose from lactose into the capsule and capsule thickness results. In exchange, we have added additional *in vitro*, *in vivo*, RNA sequencing and metabolomics data (as derived by ^{1}H-NMR) which provide more conclusive mechanistic explanations regarding the metabolization of HMOs (revised figures 1, 2, 4 , 5 and 6).

Specific points

Instead of testing CCR by using cells pre-grown on glucose or galactose before exposure to HMOs, a more informative approach would have been to grow the strain in the presence of two sugars simultaneously and monitor diauxic growth, sequential utilization of carbon sources, or changes in gene expression. While poor or absent growth after pre-growth on glucose may suggest CCR, it is not

a definitive evidence. It would also be relevant to examine whether genes involved in HMO metabolism contain cre sites in their promoters. Additionally, testing a ccpA mutant strain could have provided stronger support for CCR involvement.

Answer: We fully agree that more data is helpful to better understand the metabolization of the HMOs. Please note, we have newly added diauxic growth curves (revised Figure 1). We noted that in the presence of Glc, subsequent metabolization of the three HMOs (LNnT, 6SL and 3SL) was only observed after cells began to lyse. In contrast, continuous growth was noted in the presence of Gal. Also, we have newly added gene expression data from D39 either grown in Lactose, LNnT or 6SL received by RNA sequencing. Interestingly, we found differences for the following: Leloir/tagatose pathway, carbohydrate transporters and amino sugar metabolization (see revised Figure 2). Indeed, this data shows a clearer picture regarding the mechanism of metabolization. In addition, we added the information that the important surface-associated exoglycosidases (*bgaA*, *nanA* and *strH*) are essential for metabolization and contain cre sites in their promoters (this has been published earlier by Carvalho *et al*; [3] and the information regarding *cre* sites is added in lines 438-441). Furthermore, Carvalho *et al* have already shown that *nanA* and *nanB*, were strongly upregulated in the *ccpA* mutant. *StrH* was affected only slightly but *bgaA* was, like *nanA* and *nanB*, upregulated in the *ccpA* mutant. Please note, we have added additional data using *bgaA*, *nanA* and *strH* KO strains (lines 155-172). Taken together, we now provide a more complete picture regarding the regulation of genes required for import and metabolization of HMOs.

This reviewer acknowledges the effort invested in applying CRISPRi technology to identify genes required for HMO utilization. However, one might question whether a more classical approach: starting with a genome-wide search for glycoside hydrolases and transporters likely involved in HMO uptake and hydrolysis, followed by targeted mutagenesis, would have been more straightforward and likely to reveal key players such as the lacEFG genes.

Answer: This is a valid comment. Reviewing the results received by CRISPRi technology, the importance of the lac II operon (*lacEFG-2)* genes could have indeed been hypothesised before doing the experiments. The benefit of the CRISPRi technology is that it is unbiased and, therefore, its ability to provide quasi-comprehensive information at genome-wide scale (information added in lines 77-80). Therefore, potentially, new essential genes can be found and explored. Actually, we were somewhat surprised regarding the findings (i.e. *lacEFG-2* genes), as we originally hypothesized that it is mainly all about the exoglycosidases (*bgaA*, *nanA* and *strH*).

We also would like to stress out that the RNA sequencing and CRISPRi technology are not redundant but complementary. As an example, certain genes which were found to upregulated did not play a

major role (as shown for the *celB* and *celD* genes; see below). Therefore, by applying both and cross-validation of data, a more in-depth look into function is possible (lines 218-221).

Moreover, the CRISPRi strategy did not succeed in identifying potential transporters for 3FL and LNT, which remains a significant gap.

Answer: This situation might be explained by transporter redundancy. In this case, knocking down only one transporter gene with a single sgRNA would not affect growth/fitness. Future work, for instance applying dual CRISPRi-seq [2], may provide insights into transporter redundancy. This has now been added to the Discussion (lines 466-471). Furthermore, we have added new data regarding this issue. Namely, we newly added ^{1}H-NMR spectra of samples of post-culture chemically defined media (CDM) in order to quantify the decreasing substrates and increasing products (supplementary figure 14). The data indicates that 3FL is not consumed and we therefore hypothesize that the observed minor growth might be due to the metabolization of 'contaminating' metabolites rather than actual 3FL. Importantly, we have also newly added ^{1}H-NMR spectra for growth in LNnT and Lactose (Figure 4 and supplementary figure 13). An accumulation of Gal was observed fostering the understanding of HMO metabolization.

HMO utilization was examined, and it was shown that 2FL,despite not being metabolized by S. pneumoniae, inhibits the pathogen cellular adhesion. However, no mechanistic explanation for this observation is provided. Several HMO are known to act as decoys, preventing pathogen binding to host cells, but in the case of S. pneumoniae, the specific adhesin(s) likely targeted by 2FL or lactose have not been identified or experimentally confirmed. It was hypothesized that non metabolizable HMO, such as 2FL would show increased inhibition of bacterial adhesion to host cells, compared to metabolizable sugars such as lactose. However, no differences in inhibition were seen for 2FL and lactose in Detroit 562 cells. Why lactose was not tested in the ALI cell culture model?

Answer: The reviewer correctly points this out. We did not provide mechanistic explanations for the inhibition *of S. pneumoniae* by 2FL. Previous work revealed two newly identified non-enzymatic carbohydrate-binding modules (CBMs) of BgaA that mediate adherence to the host cell surface displayed lactose or N-acetyllactosamine [4] (newly added in lines 493-500). We hypothesize that this mechanism may also be relevant for 2FL due to structural similarities of the inhibiting molecules. Importantly, 2FL cannot be metabolised and, therefore, 2FL might be better suited as an inhibitor as compared to lactose. This conclusion can be drawn by our latest data from our in vivo model (figures 6c and 6e). Also, while we did not test lactose in the ALI cell culture model, this was newly done *in vivo* and compared to 2FL.

The manuscript mentions eIIA and eIIC genes, but their identity and functional roles are not adequately explained. Additionally, the infection experiments using the lacG mutant should have included a lacE mutant as a positive control, since previous studies have reported that lacE deletion reduces the ability to infect mice.

Answer: Sorry for the confusion as there has been a mistake. We actually meant to say that eIIA and eIIC were genes from the *celBCD* operon. It has been suggested that increased expression of *celB*, encoding a putative cellobiose-specific phosphotransferase system (PTS) IIC component might be relevant for the transport of Lac in *Lactococcus lactis* MG1363 [5]. We therefore hypothesized that this PTS system may compensate for the loss of LacE-2. We found that *celB* was upregulated in the transcriptome data but no influence on growth has been found in our study (Figure S12). This information has newly been added.

Also, we did not make very clear that the *lacE* from the Haya *et al* paper [6] **corresponds to *lacE-1* and not *lacE-2*. *lacE-1* did not show any relevance in our study and we therefore decided to delete the sentence from the manuscript to not create any confusion.**

However, a genome-wide association study revealed that variants within *lacE-2* were strongly associated with carriage isolates from children with pneumonia compared to those from healthy community-based children. We did not find that *lacG-2* had an influence on pneumococcal adherence, but if Lac or 2FL was co-inoculated with bacteria, differences were indeed noted *in vivo* (Figure 6). The effect of knocking out *lacG-2* from the lac II operon might therefore depended on the availability of oligosaccharides.

It is also unclear which components of the PTS are encoded by lacE and lacF; this should be specified. Furthermore, lacE and lacF (?) mutants were used in cell adhesion assays, yet no differences were observed compared to the wild type. The rationale for expecting a lactose transporter to influence adherence is not evident.

Answer: Yes, this has not been well written. We used a *lacE* mutant and compared to the *celB* and *celD* mutant strains. We did not use a *lacF* mutant strain. As hypothesized by the reviewer, there was indeed no difference in adherence. For a better focus, we decided to newly omit this data from the manuscript. However, our latest data including the *ΔlacG-2* and WT strains in combination with either lactose or 2FL show differences in adherence (Figure 6E). This could indicate that non-metabolization of the inhibiting agent (2FL) could indeed matter.

<u>Reviewer #3 (Remarks to the Author):</u>

In this manuscript by Bacher et al. the investigators identify and then examine the role of the PTS lactose operon during growth of S. pneumoniae in the presence of human milk oligosaccharides. This is relevant as infants, are presumably exposed to these oligosaccharides, during nursing impacting the bacterium's physiology. The methodology for included experiments is robust, however, the authors make claims that are not robustly supported by the included results. These are detailed below. Completion of the additional experiments needed to support their claims would vastly improve the manuscript.

Answer: Thank you for the encouraging comments and constructive suggestions. Please note, we added new data from additional experiments to more robustly support our claims (see below).

1) The authors claim that growth on HMO requires carbon catabolite derepression. This is supported by the observation that pre-growth of the bacteria in glucose containing media impairs Spn outgrowth in HMO-supplemented media, whereas pre growth in galactose containing media favors growth. This is circumstantial and it does not directly show that CCR must be derepressed during growth in the presence of HMO. For example, authors could measure H2O2 which is produced in greater amounts during mixed fermentation, comparing these to glucose and galactose controls. qRT-PCR for lacO, pfl and spxB would also be a way to show this.

Answer: These are great suggestions. Please note, we have newly added H_2O_2 measurements in our manuscript (Figure 4c). As expected, greater amount of H_2O_2 was observed in Gal as compared to Glc controls. Also, H_2O_2 amounts were low if grown in Lactose.

As for the HMOs, the amounts of H_2O_2 were similar as compared to Gal. Importantly, we have also newly added ^{1}H-NMR spectra for growth in Lactose (Supplementary figure S13), 3FL (Supplementary figure S14) and LNnT (Figure 4a and 4b). Regarding the latter, we also quantified the end products (lactate and formate). The increasing amounts of formate underline the hypothesis that there is mixed fermentation.

As for the genes from the pyruvate pathway (i.e. *lacO, pfl* and *spxB*), differential gene expression (lactose versus LNnT) at OD 0.4 showed Log2FC values of -0.95 (*lctO*), -1.4 (*pfl*), -1.4 (adh) and -0.04 (*spxB*) indicating the *pfl, adh* and *lctO* are indeed upregulated (in LNnT) (supplementary table 4). We added this new information related to the additional transcriptome data to the manuscript (lines 326-329).

2) Along such lines, the authors should show that the lac operon is also expressed at higher levels during growth conditions associated with CCR derepression.

Answer: This is an interesting point. Our transcriptome data shows a very high upregulation of the lacI operon genes (*lacABC*) but not lacII (*lacEFG2*) operon if grown in LNnT and 6SL (supplementary tables 4-8). In contrast, the CRISPRi data clearly indicate the essentiality of the latter. This underlines the fact that expression and CRISPRi data are showing different aspects of regulation and are, therefore, complimentary (information added in lines 220-221).

3) Its also unclear if on line 241 if the authors mean that 81-92% of all the labeled carbon became associated with polysaccharide. Or if instead 81-92% of de novo capsule was made of labeled sugars. Does polysaccharide mean just capsule or capsule, teichoic acid, peptidoglycan, all of which are polysaccharides?

Answer: Please note, to make the manuscript more focused (see comment of reviewer 2), we have decided to omit the capsule labelling and thickness data. Still, we meant to say that 81-92% of de novo capsule was made of labelled sugars

4) Give than lac metaboization is dispensable during in vivo experimentation it is suprising that the authors did not reexamine their postulated model that HMOs may find their way to the respiratory tract and this may be a boon for bacteria that can degrade these carbon sources. Without this finding the rationale proposed for the paper and title seems to be unmerited. Perhaps examining colonization levels in mice that are instilled intranasally with HMOs is a way to show that the WT can outgrow the lac operon mutant under these conditions? Alternatively use a nursing pup model of colonization to test this.

Answer: We have added new data showing colonization levels in mice that were instilled intranasally with either lactose or 2FL (Figures 6C and 6D). We found that if instilled with 2FL, more inhibition could be seen as compared to lactose for the WT D39.

Also, we could indeed confirm that the WT outgrew the *ΔlacG-2* strain if co-inoculated with lactose (though the difference was small) (Figure 6E).

5) The adhesion experiment to cell lines were done using R6 and not D39. R6 is 100-fold more adhesive than D39 and this may obfuscate important results.

Answer: We agree that adherence data for D39 is important, too. This can newly be seen in Figure 5A and we could confirm the inhibitory effect of lactose and 2FL. As pointed out correctly by the reviewer, adherence of D39 was around 100-fold less as compared to R6. This information has also been added (lines 356-359).

6) The authors show that 2FL blocks adherence in vitro, but not necessarily how. These experiments should be repeated using the lac operon deficient mutant so that at least the transporter itself can be ruled in/out.

Answer: Please note, we did the suggested experiment (on Detroit cells) and found indeed (again) inhibition for the lac operon deficient mutant strain. In addition, we used the deficient mutant strain (in combination with 2FL) in our in vivo model. The latter is added in figure 6. As for the in vitro data, we decided not to include it to avoid making the paper too dense.

Regarding how the adherence works, previous work revealed two newly identified non-enzymatic carbohydrate-binding modules (CBMs) of BgaA that mediate adherence to the host cell surface displayed lactose or N-acetyllactosamine [4] (lines 493-498). We hypothesize that this may also be the case for 2FL due to structural similarities of the inhibiting molecules. However, 2FL cannot be metabolised and, therefore, might be better suited as an inhibitor. This has now been better clarified in the revised manuscript.

7) Again, and given the lack of in vivo validation, the authors should consider treating colonized mice with 2FL and determining if this impacts colonization burden.

Answer: See answer to point 4) above. The experiments with 2Fl have been done.

References

1. Kobata, A., *Exo- and endoglycosidases revisited.* Proc Jpn Acad Ser B Phys Biol Sci, 2013. **89**(3): p. 97-117.
2. Denereaz, J., et al., *Dual CRISPRi-seq for genome-wide genetic interaction studies identifies key genes involved in the pneumococcal cell cycle.* Cell Syst, 2025. **16**(11): p. 101408.
3. Carvalho, S.M., et al., *CcpA ensures optimal metabolic fitness of Streptococcus pneumoniae.* PLoS One, 2011. **6**(10): p. e26707.
4. Singh, A.K., et al., *Unravelling the multiple functions of the architecturally intricate Streptococcus pneumoniae beta-galactosidase, BgaA.* PLoS Pathog, 2014. **10**(9): p. e1004364.
5. Solopova, A., et al., *A specific mutation in the promoter region of the silent cel cluster accounts for the appearance of lactose-utilizing Lactococcus lactis MG1363.* Appl Environ Microbiol, 2012. **78**(16): p. 5612-21.
6. Hava, D.L. and A. Camilli, *Large-scale identification of serotype 4 Streptococcus pneumoniae virulence factors.* Mol Microbiol, 2002. **45**(5): p. 1389-406.

<u>Point-by-point response to the reviewers' comments</u>

<u>Reviewer #1 (Remarks to the Author):</u>

No comments have been given

<u>Reviewer #2 (Remarks to the Author):</u>

The revised manuscript has been substantially improved, including additional experiments and removing data that somewhat detracted from the main focus (e.g., sugar incorporation into the capsule). My primary concern in the original version was the lack of mechanistic insights. While the possibility that HMOs are present in the upper airways of breastfed infants remains speculative and is not directly demonstrated, the authors now provide new in vivo evidence in mice showing that lactose and 2FL affect the persistence of S. pneumoniae strains. In this regard, it should be noted that lactose is not an HMO, and this should be corrected in Figure 6.

Answer: This is a valid point. We corrected that lactose is not an HMO in the text.

Concerning CCR during HMO utilization, the authors have added new growth curves and RNA-seq analyses of gene expression, providing a more comprehensive understanding of HMO metabolism in S. pneumoniae. In addition, several inaccuracies and insufficiently explained points from the previous version have been addressed. Despite these improvements, I still believe that the most significant finding of the study, namely that HMOs may inhibit S. pneumoniae colonization, remains insufficiently explored at the mechanistic level. The work is technically sound and carefully executed, but it largely remains descriptive. Specifically, the manuscript identifies genes involved in HMO utilization through CRISPRi-seq, characterizes their transcriptional regulation in response to different HMO, examines CCR in the presence of glucose (growth curves), and demonstrates that the non-metabolizable HMO (2FL) reduces bacterial adhesion in vitro and pneumococcal carriage in a mouse model. However, the molecular basis of this inhibitory effect is not investigated. It remains unclear which pneumococcal adhesins or other bacterial factor(s) may mediate the observed reduction in adhesion and colonization. Likewise, the potential impact of metabolizable HMOs on colonization (inhibitory or stimulatory) is not addressed.

Answer: We agree that the molecular basis of the inhibitory effect needs more work. However, it has been hypothesized that BgaA might be relevant if lactose is used as an inhibiting compound [1]. Considering the tested HMOs have structural similarities compared to lactose, an influence of BgaA on inhibition seems an interesting hypothesis. Also, we indeed did not test metabolizable HMOs but used metabolizable lactose instead. Related to this issue, please also note our reply below.

The manuscript covers two distinct topics: the genetic basis of HMO utilization and the effect of HMO on S. pneumoniae adhesion and colonization. However, neither aspect provides sufficiently conclusive or mechanistic insights.

For example:

The observation that the PTS transporter encoded by lacEF is not essential for LNT utilization is not adequately explained. This transporter is required for the utilization of other HMOs such as LNnT, 3SL and 6SL. According to the model presented in Figure 7, all tested HMO are processed by extracellular glycosidases, releasing lactose, which is then imported through LacEF. If LNT metabolism also generates lactose, why does it differ from the other HMO in its dependence on LacEF? This apparent inconsistency requires clarification.

Answer: Many thanks for this comment. The reviewer is right that we cover two distinct topics in our article. We feel that we address the basis of HMO quite comprehensively but, indeed, open questions remain; especially regarding the mechanisms of the observed reduction in adhesion and colonization. Regarding LNT, we agree that this has not been clearly described in our article. We meant to say that we found no evidence that BgaC is involved in the metabolization of LNT, in contrast to an earlier report (therefore this is illustrated with a 'dashed' arrow in figure 7) [2]. Perhaps, this lack of confirmation is a result of the chosen culture conditions. Therefore, we hypothesize a similar scenario for LNT as 3-FL. That is, a) that this situation might be explained by transporter redundancy. In this case, knocking down only one transporter gene with a single sgRNA would not affect growth/fitness. Future work, for instance applying dual CRISPRi-seq [3], may provide insights into transporter redundancy. Alternatively, b) the observed minor growth in the presence of high concentrations of LNT could also be due to 'contaminating' metabolites rather than actual LNT. Please note, we made this clearer in the manuscript.

Could certain HMO serve as readily available carbon sources that enhance bacterial growth and persistence in vivo? The animal experiments focus exclusively on the inhibitory HMO 2FL. However, several HMO are efficiently metabolized by S. pneumoniae. Testing whether these compounds promote growth, colonization, or persistence in the mouse model would be highly informative and would provide a more balanced assessment of the biological consequences of HMO utilization.

Answer: Thanks for this important feedback. Indeed, our animal experiments focus on the inhibitory HMO 2'FL. The reason for choosing 2'FL was mainly due to the results of the adherence assays (Figure 5) which suggested that 2'FL could be a promising candidate for the animal experiments. We agree that testing the other compounds would also be highly informative but would like to point out that we used lactose which, like some of the HMOs e.g. LNnT and 6'SL, is efficiently metabolized by

***S. pneumoniae.* In addition, lactose has been identified as a promising compound for the inhibition of colonisation in our adherence experiments (Figure 5) but also in a previous study [1]. Interestingly, lactose did not prove to be an efficient inhibitor in our mouse model unless we used the KO strain unable to metabolize lactose. Taken together, our results provide a starting point for further investigations and we agree that more testing is necessary to better understand colonization and biological consequences of HMO utilization.**

<u>Reviewer #3 (Remarks to the Author):</u>

The authors have addressed my concerns. This is a very nice manuscript.

Answer: Many thanks for this. This is very encouraging feedback.

References

1. Singh, A.K., et al., *Unravelling the multiple functions of the architecturally intricate Streptococcus pneumoniae beta-galactosidase, BgaA.* PLoS Pathog, 2014. **10**(9): p. e1004364.
2. Jeong, J.K., et al., *Characterization of the Streptococcus pneumoniae BgaC protein as a novel surface β-galactosidase with specific hydrolysis activity for the Galβ1-3GlcNAc moiety of oligosaccharides.* Journal of bacteriology, 2009. **191**(9): p. 3011-3023.
3. Denereaz, J., et al., *Dual CRISPRi-seq for genome-wide genetic interaction studies identifies key genes involved in the pneumococcal cell cycle.* Cell Syst, 2025. **16**(11): p. 101408.
