# Supplementary material for: Metabolization of human milk oligosaccharides by Streptococcus pneumoniae
Source: Nat Commun. 2026 Sep 30;17:11242. doi: 10.1038/s41467-026-77970-7 (PMC13720028; doi:10.1038/s41467-026-77970-7)
Supplement: Supplementary file 3 [file 41467_2026_77970_MOESM1_ESM.pdf]

Supplementary figures

Supplementary figure 1:

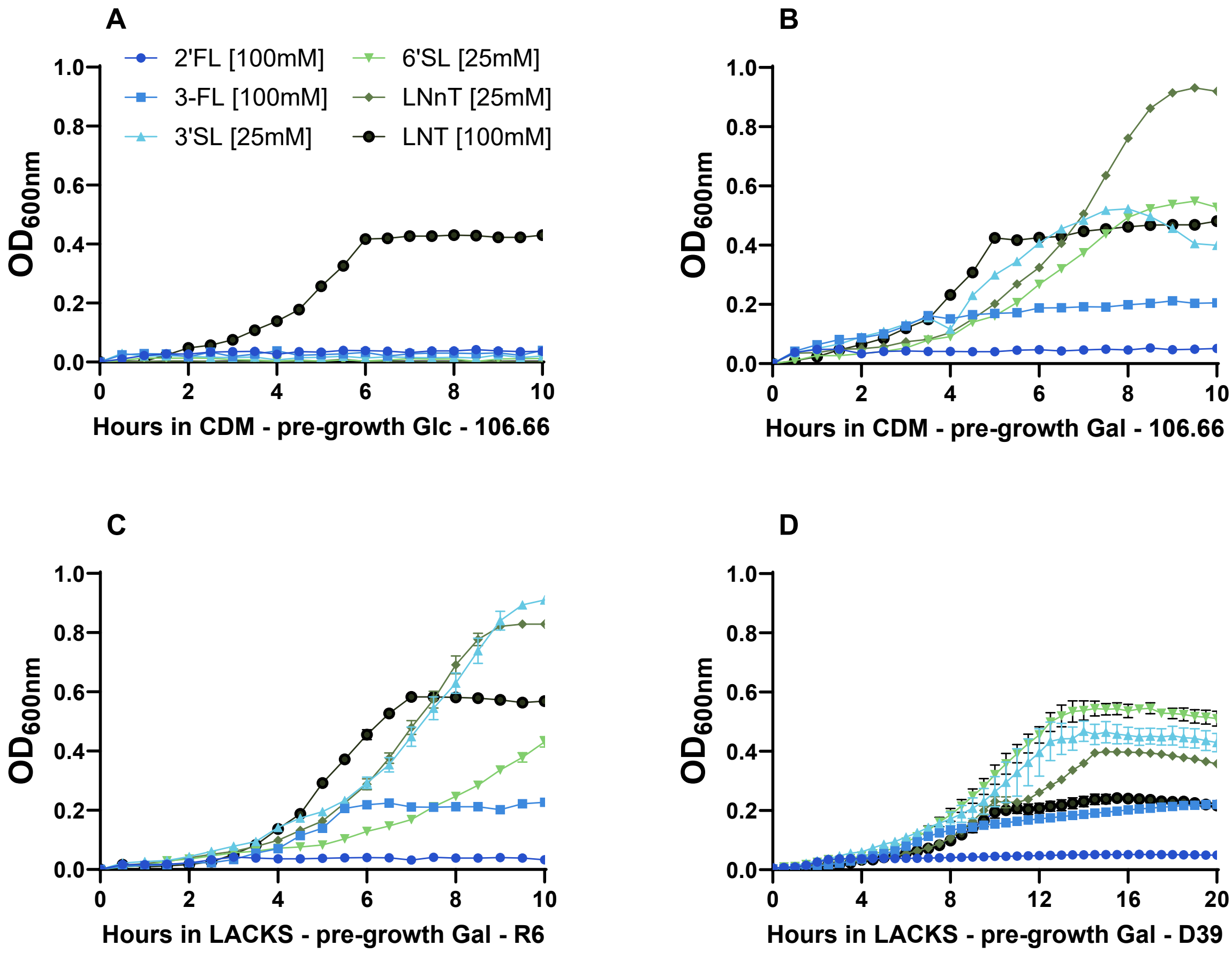

Figure S1: Pneumococcal growth curves of strains 106.66 (serotype 6B), R6 and D39 in human milk oligosaccharides (HMOs). In **A)**, the growth of 106.66 in chemically defined medium (CDM) with pre-growth in Glucose (Glc) and subsequent growth in different HMOs (2'-Fucosyllactose (2'FL), 3-Fucosyllactose (3-FL), 3'-Sialyllactose (3'SL), 6'-Sialyllactose (6'SL), Lacto-N-neotetraose (LNnT), and Lacto-N-tetraose (LNT)), each used as the sole C-source, is displayed. In **B)**, the growth of 106.66 in CDM medium with pre-growth in Galactose (Gal).

In **C)** and **D)** the growth of R6 and D39 in Lacks medium with pre-growth in Galactose (Gal) are illustrated. As D39 did not properly grow in CDM, a semi-defined medium (Lacks medium) has been chosen for the investigation of growth.

Supplementary figure 2:

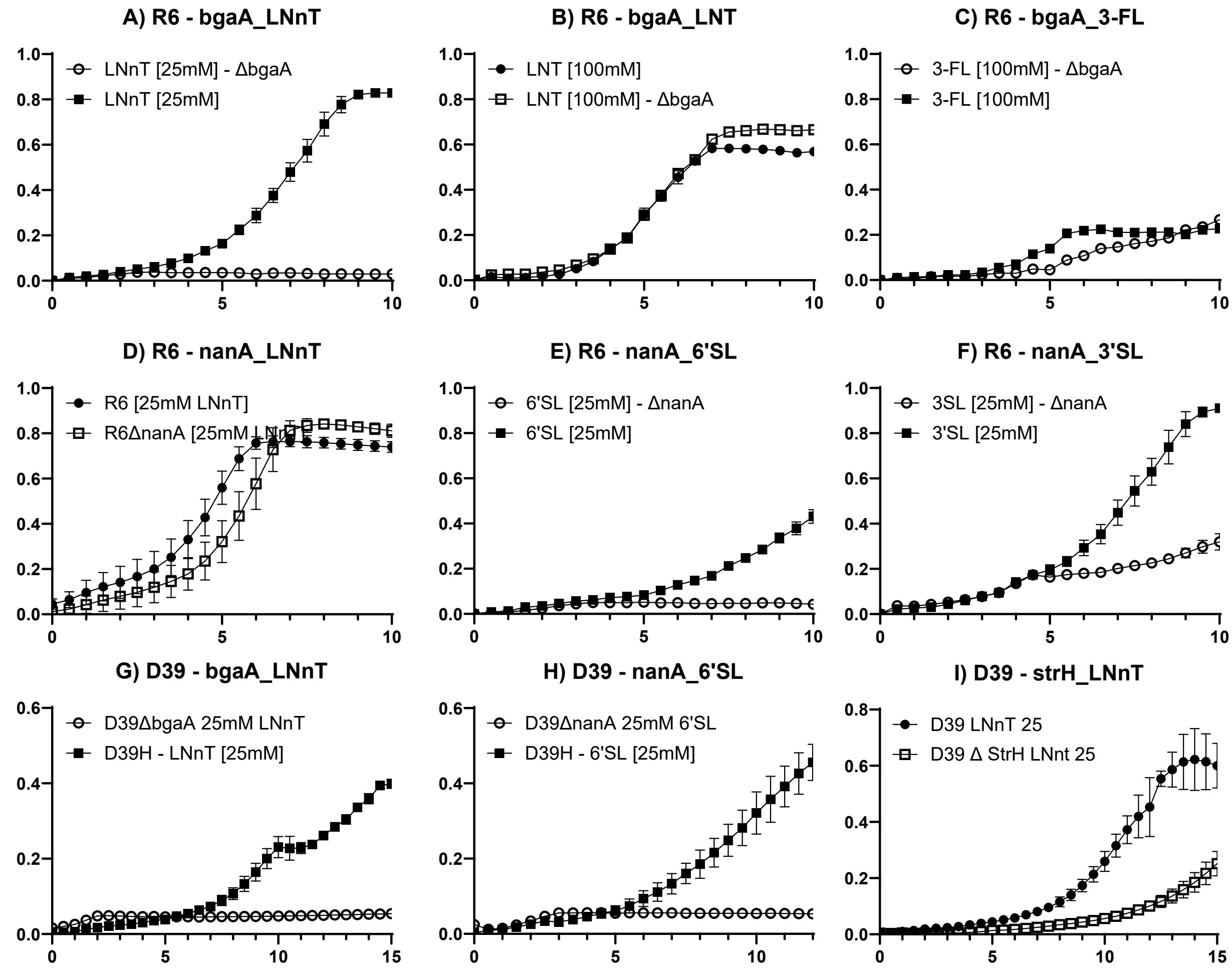

Figure S2: Pneumococcal growth curves comparing the wild-type strains D39 and R6 with the gene deletion strains containing Δ*bgaA,* Δ*nanA* and Δ*strH* in Lacks medium.

strH, *bgaA* and *nanA* mutant strains underline the importance of these surface-associated exoglycosidases in the metabolization of HMOs. The mean of two to three representative technical replicas from a single biological replicate is blotted ± SEM.

Supplementary figure 3:

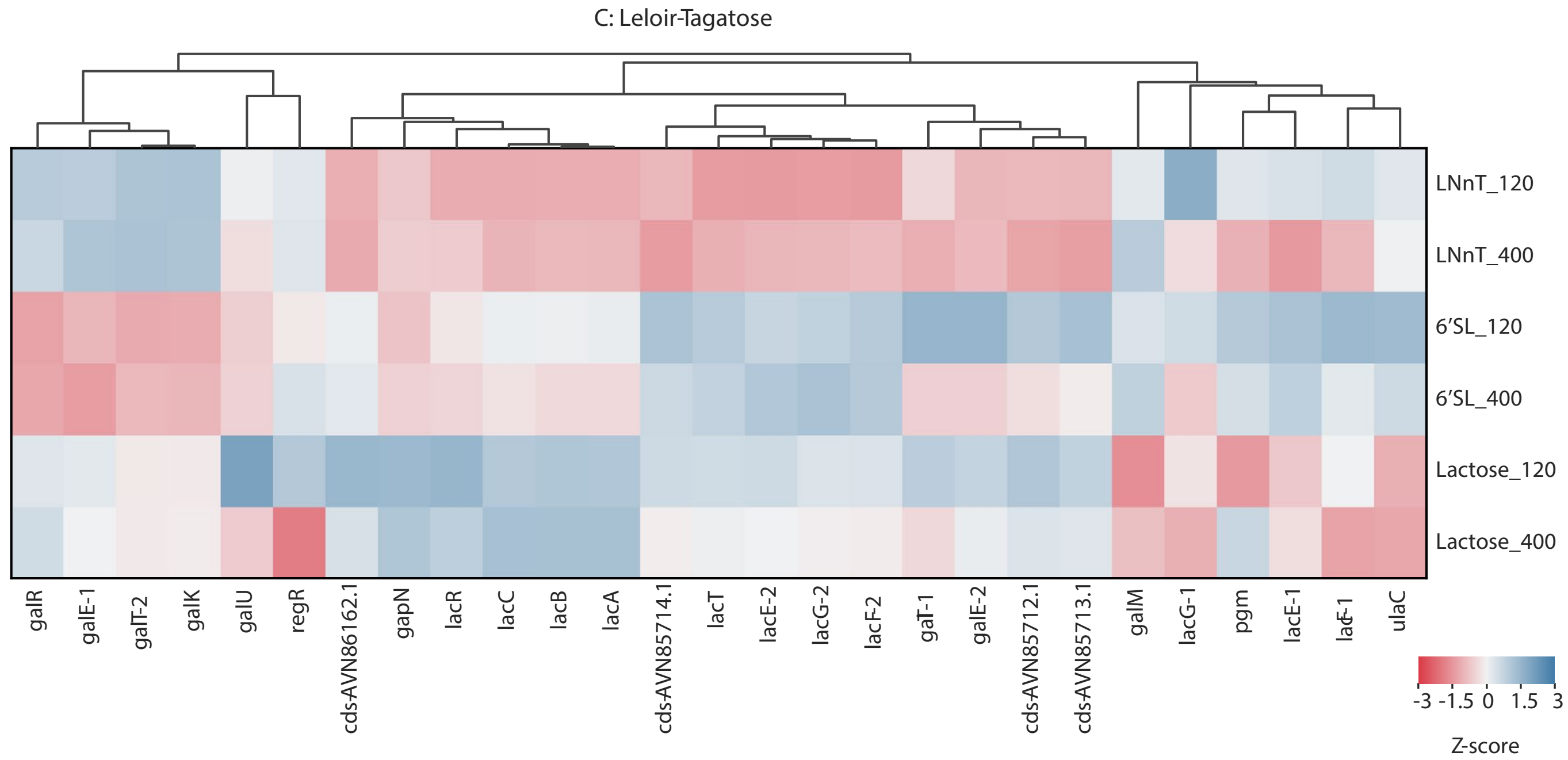

Figure S3: RNA-seq reveals expression differences for LNnT and 6'SL as compared to lactose (Lac) for Leloir and Tagatose-6P-pathway genes

Heatmap with varying z-scores of genes belonging to Leloir and Tagatose-6P-pathways are shown. Source data are provided in supplementary data 3, 4, 5 and 6.

Supplementary figure 4:

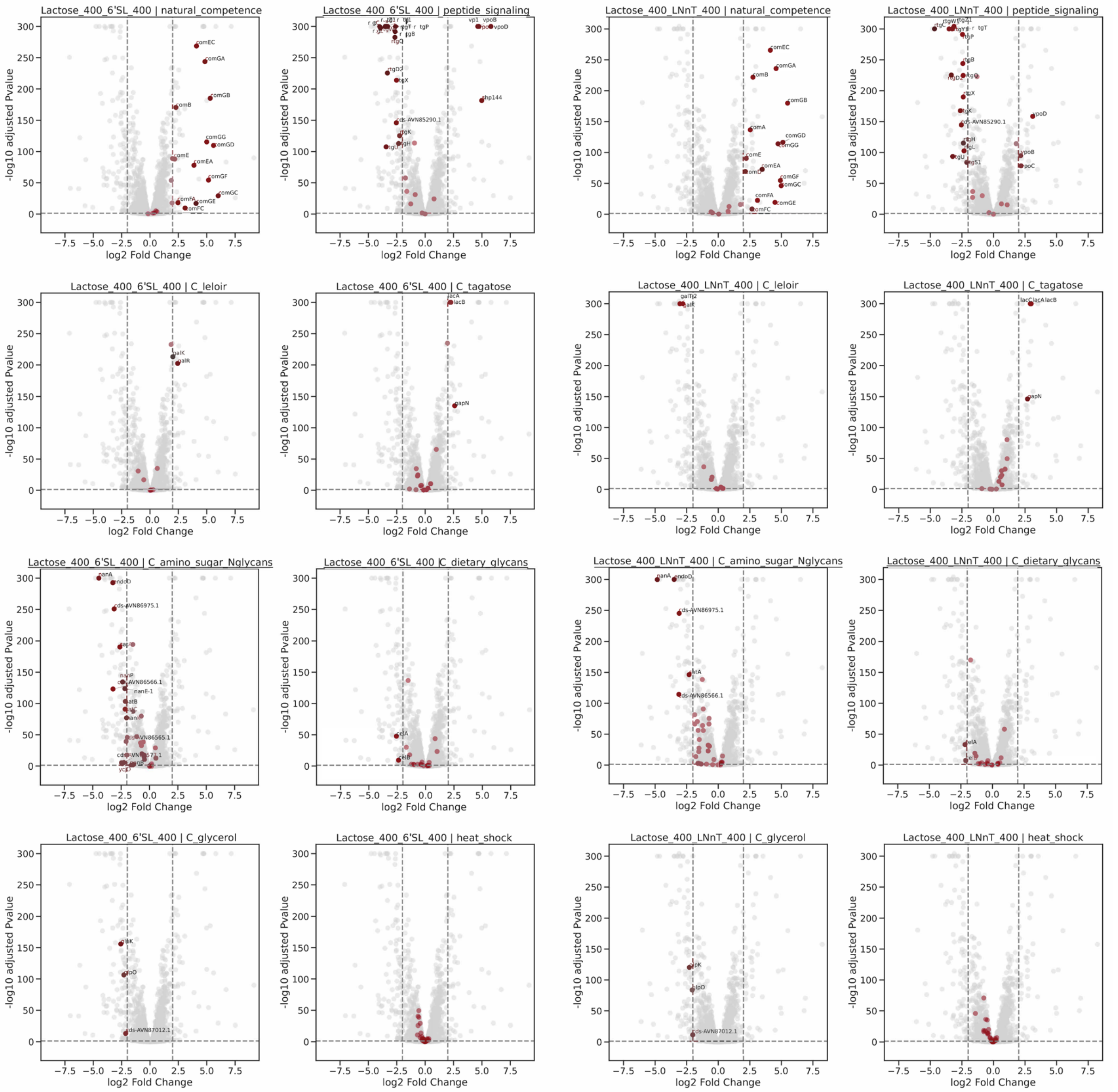

Figure S4: Volcano plots of DEGs for samples grown to OD_{600nm}=0.4.

Volcano plots of DEGs between D39 grown in Lactose and 6'SL or LNnT up to OD_{600nm} of 0.4 are illustrated. Genes of different pathways are colored and labelled if $\log_2$ fold changes were below -2.0 or above 2.0 (dotted lines). Differential enrichment was tested using DESeq2 (negative binomial generalized linear model with a Wald test) with two-tailed tests and *P*-values adjusted for false discovery rate. Source data are provided in supplementary data 3 and 4.

Supplementary figure 5:

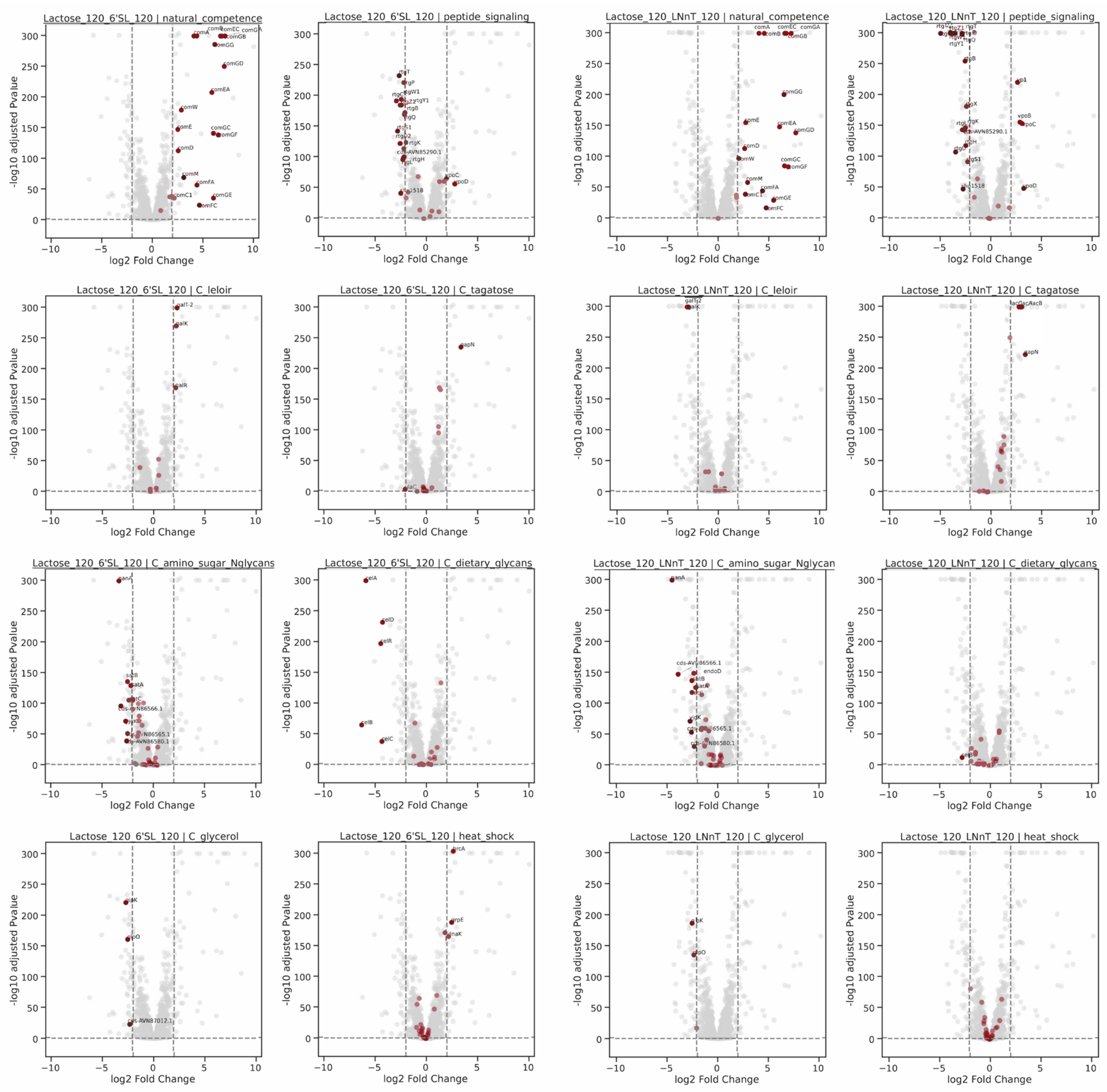

Figure S5: Volcano plots of DEGs for samples grown to OD_{600nm}=0.12.

Volcano plots of DEGs between D39 grown in Lactose and 6'SL or LNnT up to OD_{600nm} of 0.12 are illustrated. Genes of different pathways are colored and labelled if log_2 fold changes were below -2.0 or above 2.0 (dotted lines). Differential enrichment was tested using DESeq2 (negative binomial generalized linear model with a Wald test) with two-tailed tests and *P*-values adjusted for false discovery rate. Source data are provided in supplementary data 5 and 6.

Supplementary Figure 6

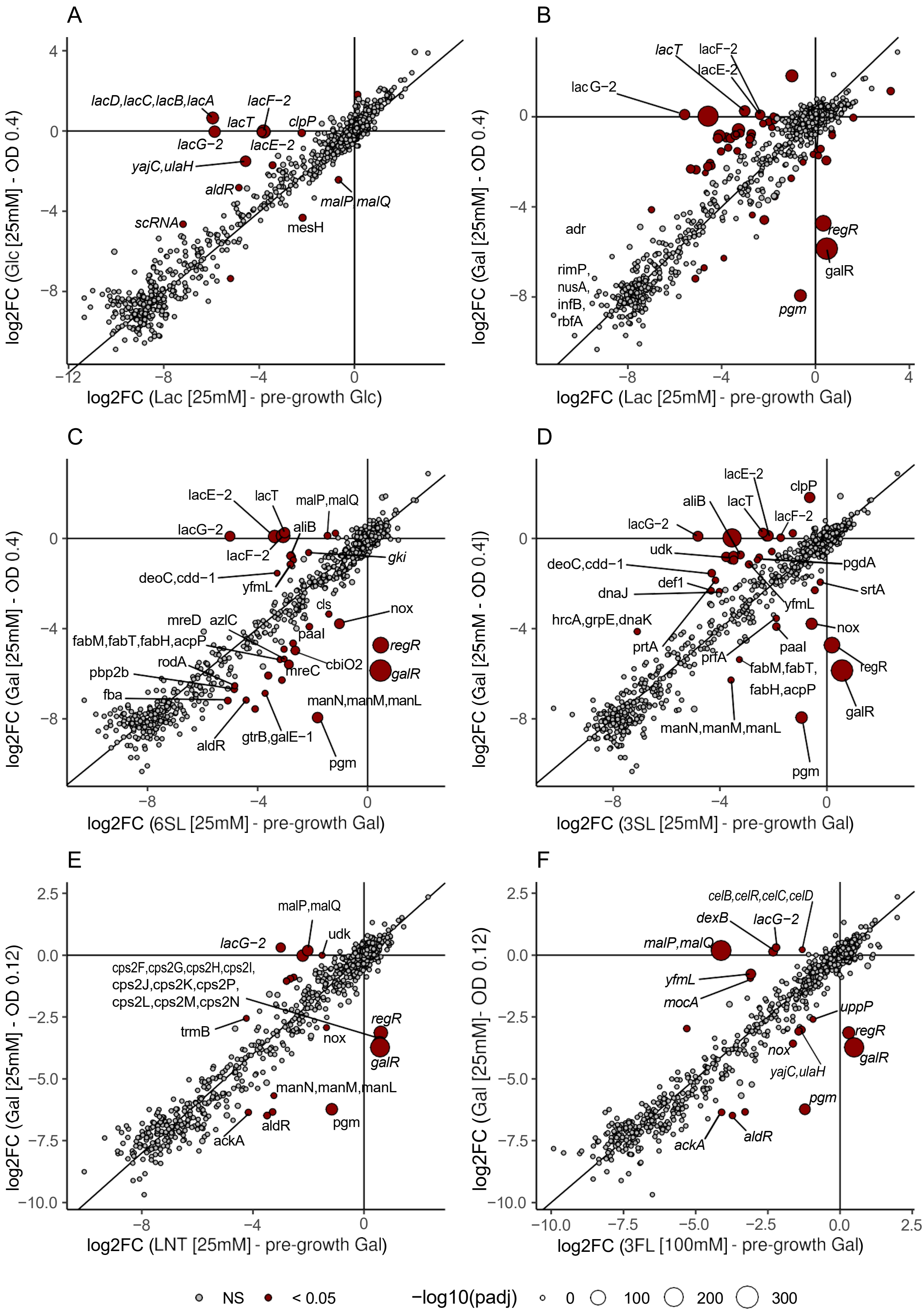

Figure S6: Genome-wide CRISPRi profiling identifies the essentiality of the *lacEF-2* operon for the metabolization of human milk oligosaccharides (HMOs) in D39 (HL23-1). In **A-B)**, the gene essentiality in Lactose (Lac) was compared to either Glucose (Glc) or Galactose (Gal). In **C-F)**, the HMOs: **C)** 6'-Sialyllactose (6'SL), **D)** 3'-Sialyllactose (3'SL), **E)** Lacto-N-tetraose (LNT), and **F)** 3-Fucosyllactose (3-FL) are compared to Gal. The *lacEF-2* operon is important for Lac (A), 6'SL (C), and 3'SL (D), while *lacG-2* was found to be vital for all tested HMOs. Log_2 fold changes (log_2FC) show fitness effects measured as depletion or enrichment of corresponding sgRNAs in the pool upon induction, and adjusted p-values are shown on an inverse log_{10} scale. Genes with p_{adj} < threshold_alpha are highlighted in red. Not all gene-hits are shown as the displayed cut-off was set at a FC of 1 and padj<0.05. Source data are provided in supplementary data 7.

Supplementary Figure 7

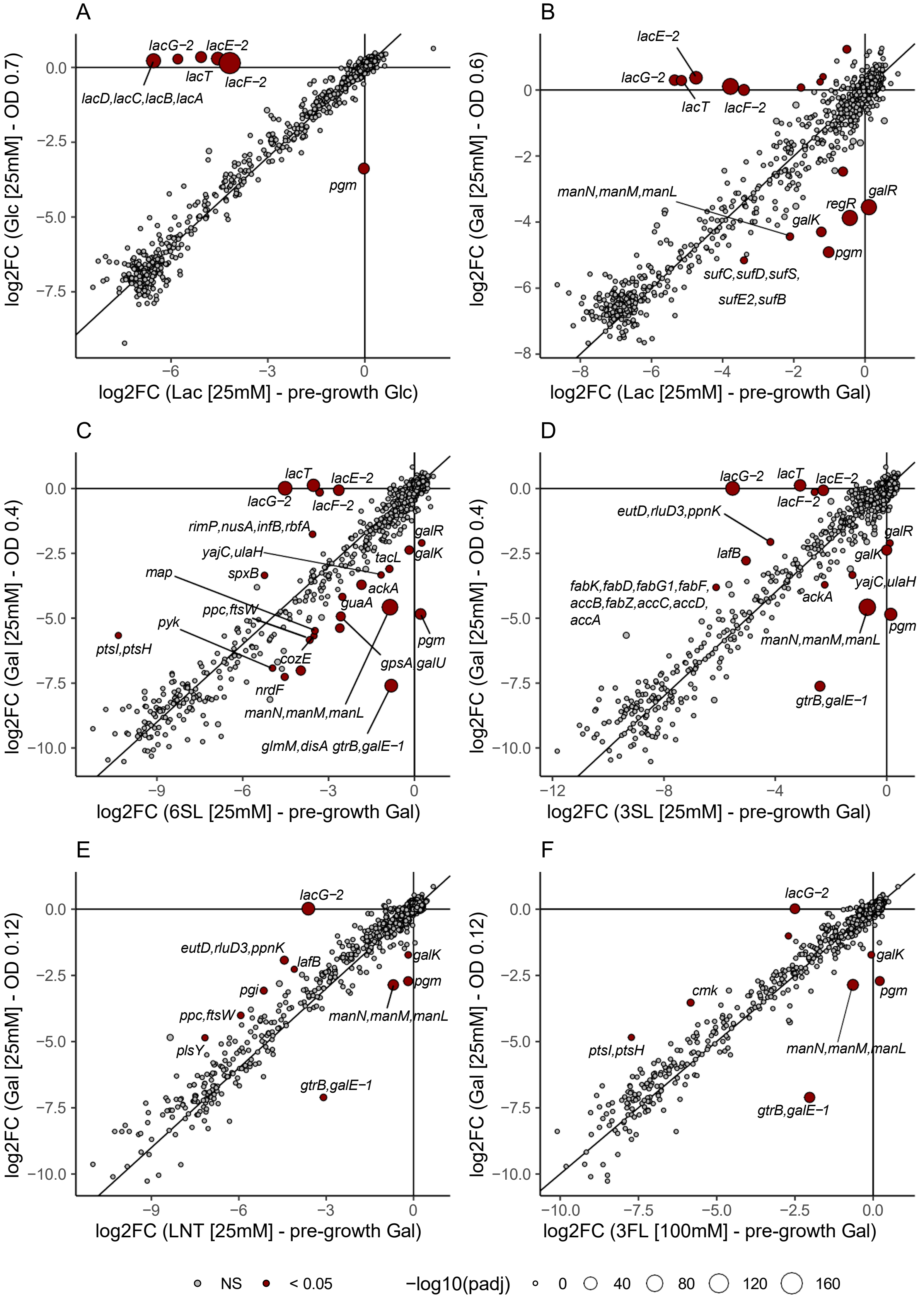

Figure S7: Genome-wide CRISPRi profiling identifies the essentiality of the *lacEF-2* operon for the metabolization of human milk oligosaccharides (HMOs) in R6 (HL23-2). In **A-B),** the gene essentiality in Lactose (Lac) was compared to either Glucose (Glc) or Galactose (Gal). In **C-F),** the HMOs: **C)** 6'-Sialyllactose (6'SL), **D)** 3'-Sialyllactose (3'SL), **E)** Lacto-N-tetraose (LNT), and **F)** 3-Fucosyllactose (3-FL) are compared to Gal. The *lacEF-2* operon is important for Lac (A), 6'SL (C), and 3'SL (D), while *lacG-2* was found to be vital for all tested HMOs. Log_2 fold changes (log_2FC) show fitness effects measured as depletion or enrichment of corresponding sgRNAs in the pool upon induction, and adjusted p-values are shown on an inverse log_{10} scale. Genes with padj < threshold_alpha are highlighted in red. Not all gene hits are shown as the displayed cut-off was set at a FC of 1 and padj<0.05. Source data are provided in supplementary data 8.

Supplementary Figure 8

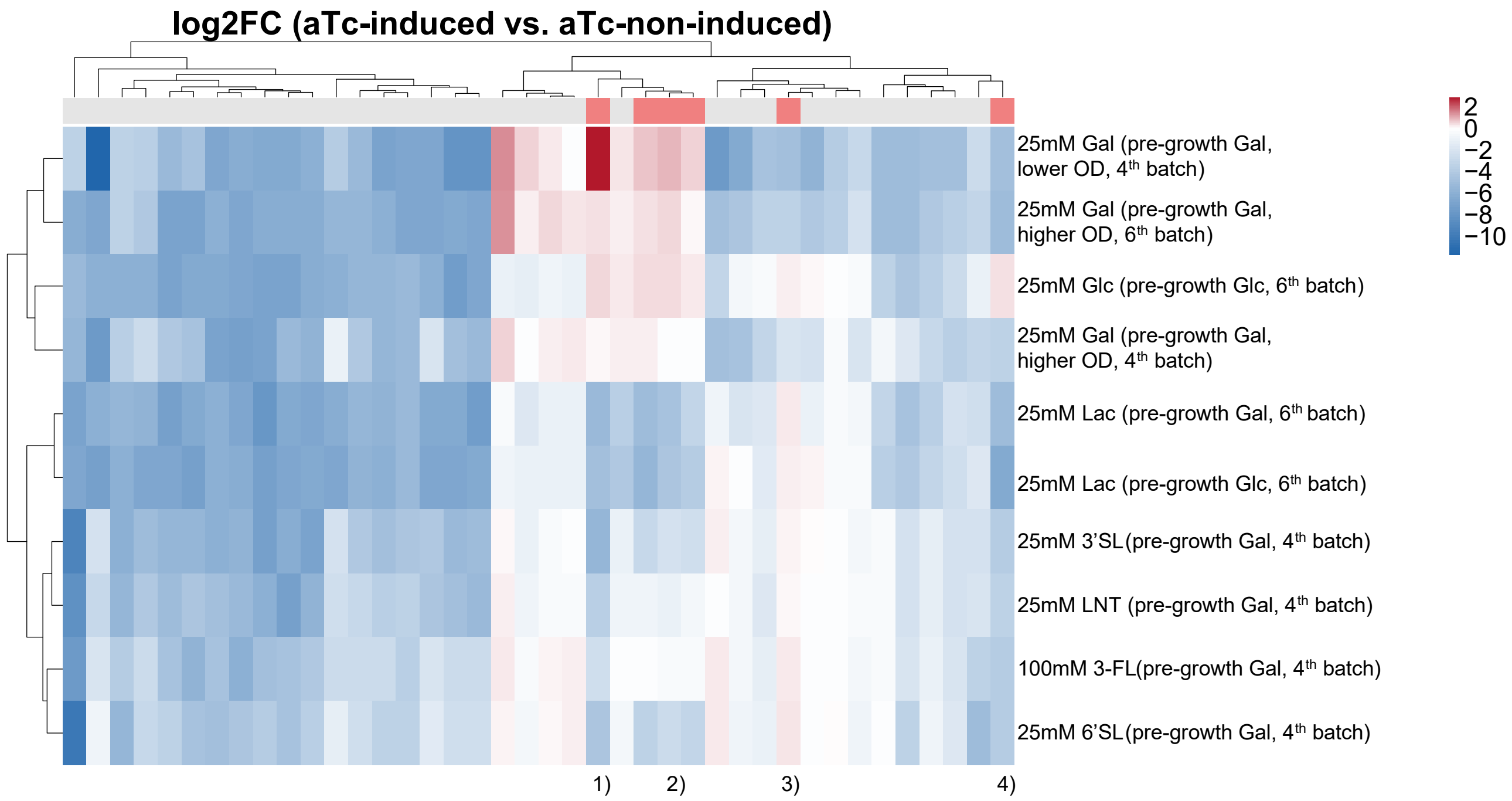

Figure S8: Heat map of all CRISPRi strains (background R6; HL23-2) illustrating differentially enriched operons. Different carbon sources were used for growth: Glucose (Glc), Galactose (Gal), Lactose (Lac), and human milk oligosaccharides (HMOs: 3-Fucosyllactose (3-FL), 3'-Sialyllactose (3'SL), 6'-Sialyllactose (6'SL), and Lacto-N-tetraose (LNT)). For strains in Gal, two different sets of replicas are shown (high and low OD), as well as two batches (4th and 6th). Important genes (1) *lacG-2*, 2) *lacT*, *lacEF-2*, 3) *galR*, 4) *lacABCD*) showing differences between strains grown in Gal as compared to the HMOs and Lac are indicated. Source data are provided in supplementary data 7 and 8.

Supplementary figure 9: LacG-2 (6-phosphate-beta-galactosidase) is essential for the metabolization of human milk oligosaccharides (HMOs).

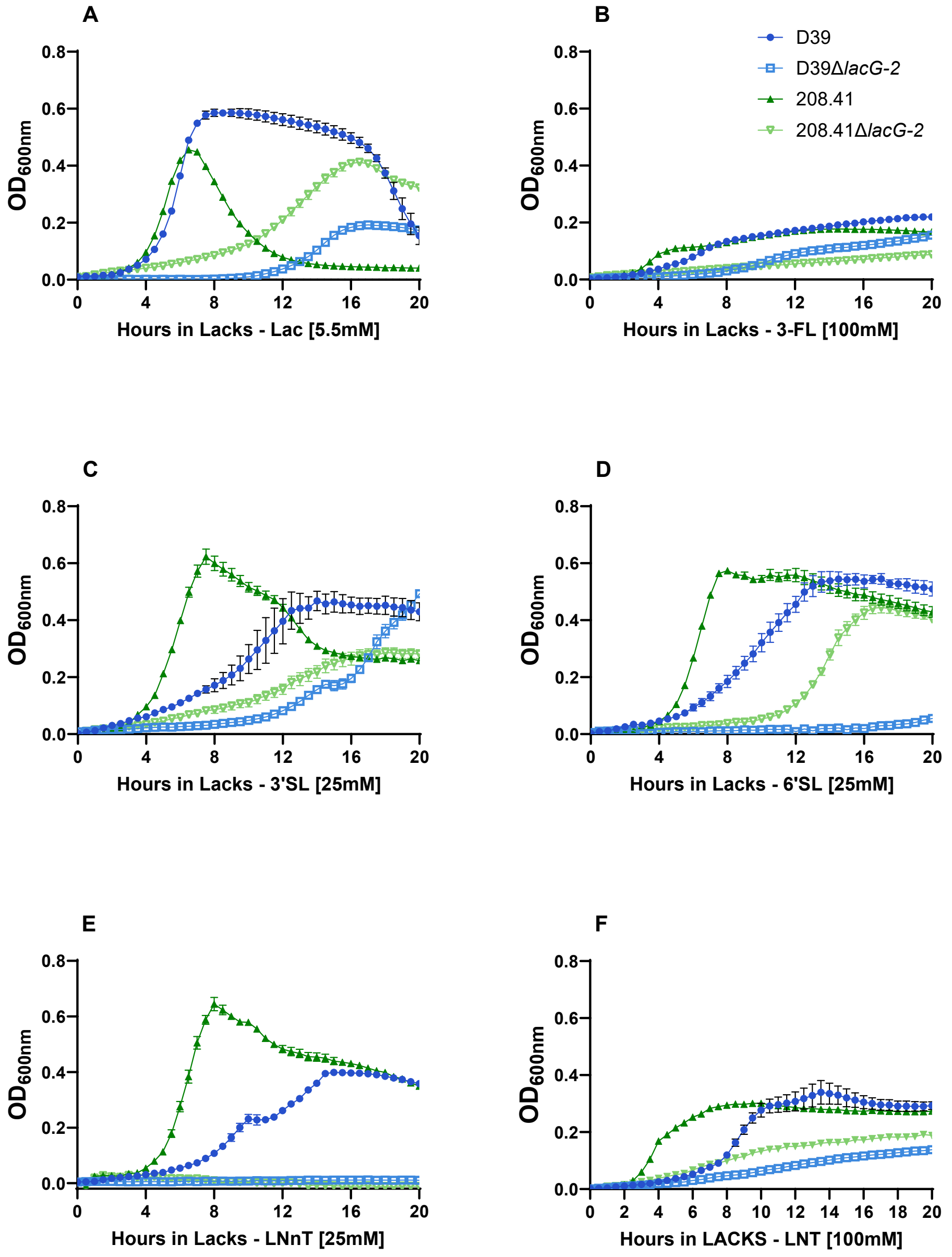

Figure S9: LacG-2 (6-phosphate-beta-galactosidase) is essential for the metabolization of human milk oligosaccharides (HMOs). Two wild-type strains, D39 and 208.41, and two *lacG-2* deletion strains, D39Δ*lacG-2* and 208.41Δ*lacG-2,* were grown in Lacks media. Each graph displays the usage of one sugar as the sole carbon source: **A)** Lactose (Lac), **B)** 3-Fucosyllactose (3-FL), **C)** 3'-Sialyllactose (3'SL), **D)** 6'-Sialyllactose (6'SL), **E)** Lacto-N-neotetraose (LNnT), and **F)** Lacto-N-tetraose (LNT). From three biological replicas, three representative technical replicas from a single biological replicate are plotted ± SEM.

Supplementary Figure 10

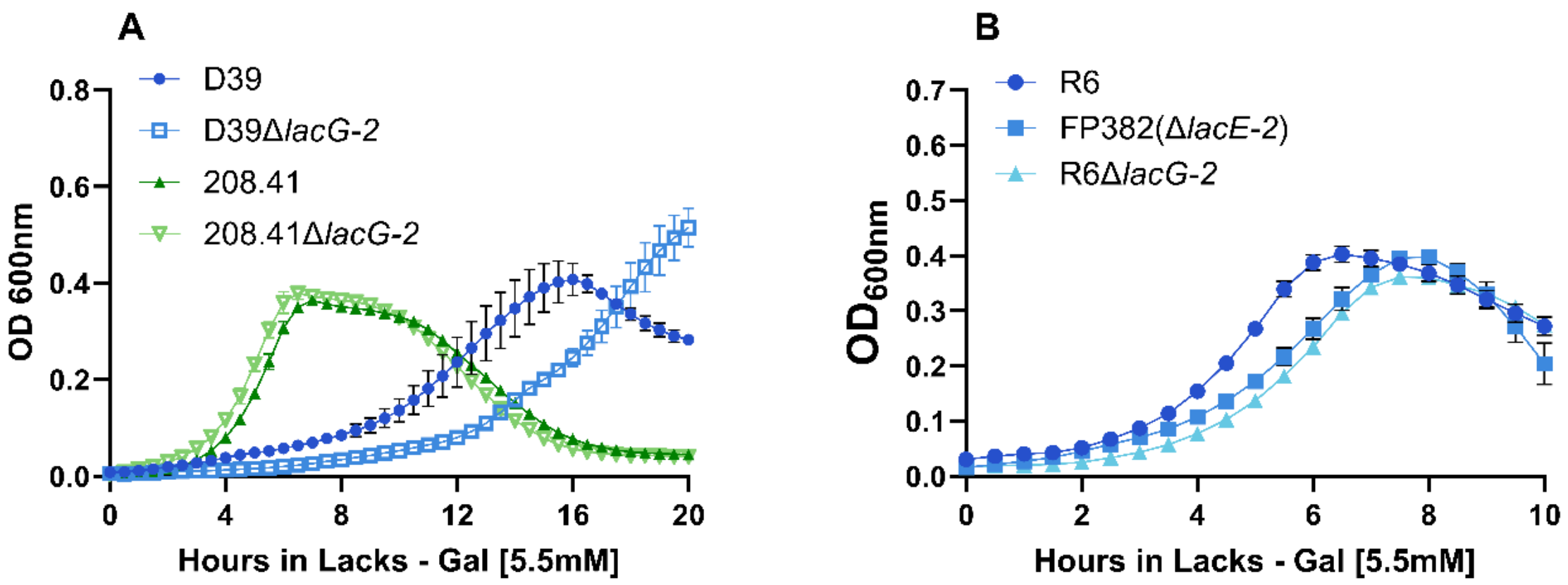

Figure S10: Pneumococcal growth curves comparing the wild-type strains D39, 208.41, and R6 with the gene deletion strains, FP382 (Δ*lacE-2*) and Δ*lacG-2* in Lacks medium. Both graphs show growth in Galactose (Gal), indicating that there is no difference, or only a minor difference, between wild-type strains and gene deletion strains when grown in Gal. The mean of two to three representative technical replicas from a single biological replicate is blotted ± SEM.

Supplementary Figure 11

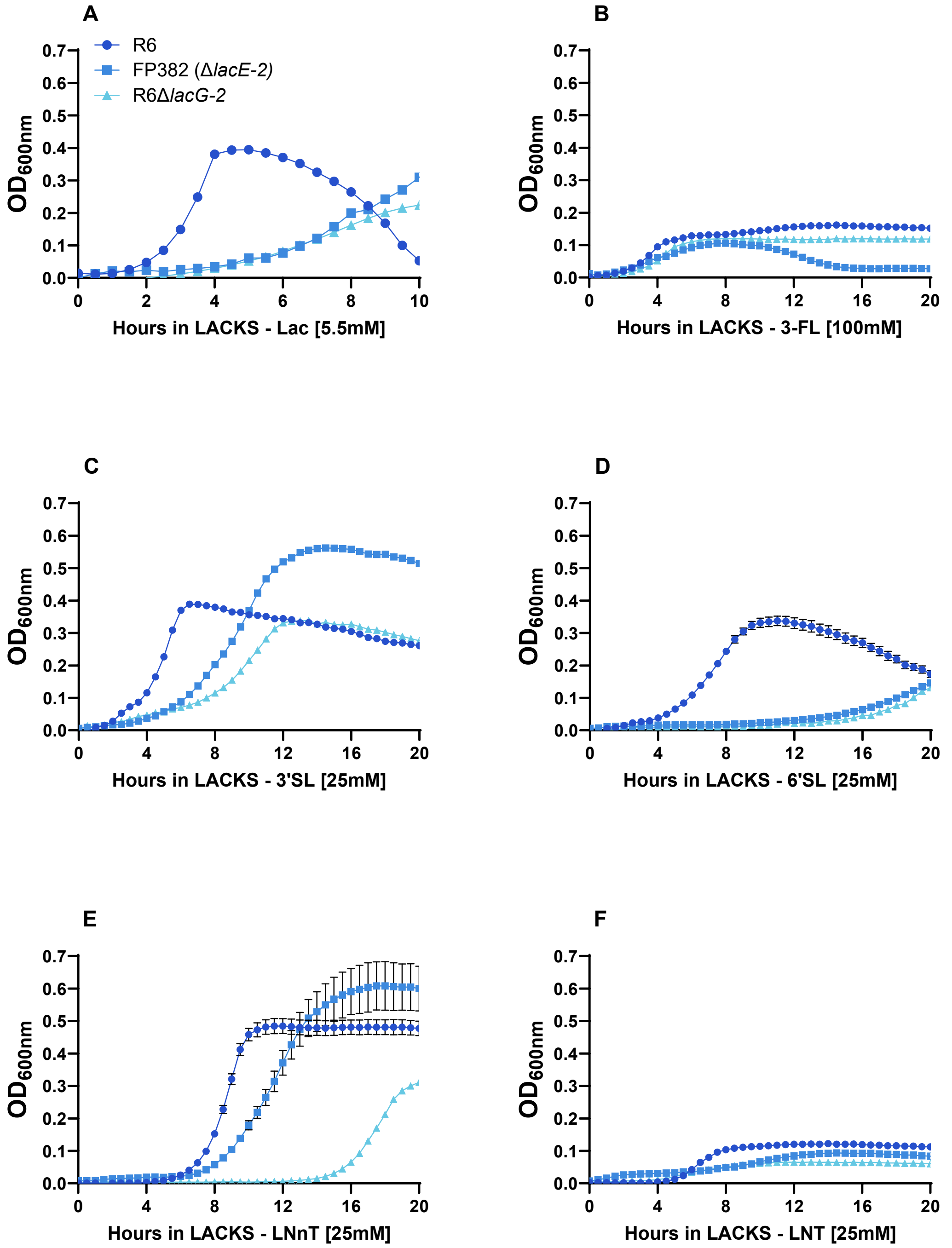

Figure S11: Pneumococcal growth curves comparing the strains R6 with the gene gelation strains, FP382 (Δ*lacE-2*) and R6Δ*lacG-2* in Lacks medium. In **A)** Lactose (Lac), in **B)** 3-Fucosyllactose (3-FL), in **C)** 3'-Sialyllactose (3'SL), in **D)** 6'-Sialyllactose (6'SL), in **E)** Lacto-N-neotetraose (LNnT), and in **F)** Lacto-N-tetraose (LNT) were used as the sole carbon source. The mean of three representative technical replicas from a single biological replicate is blotted ± SEM.

Supplementary figure 12:

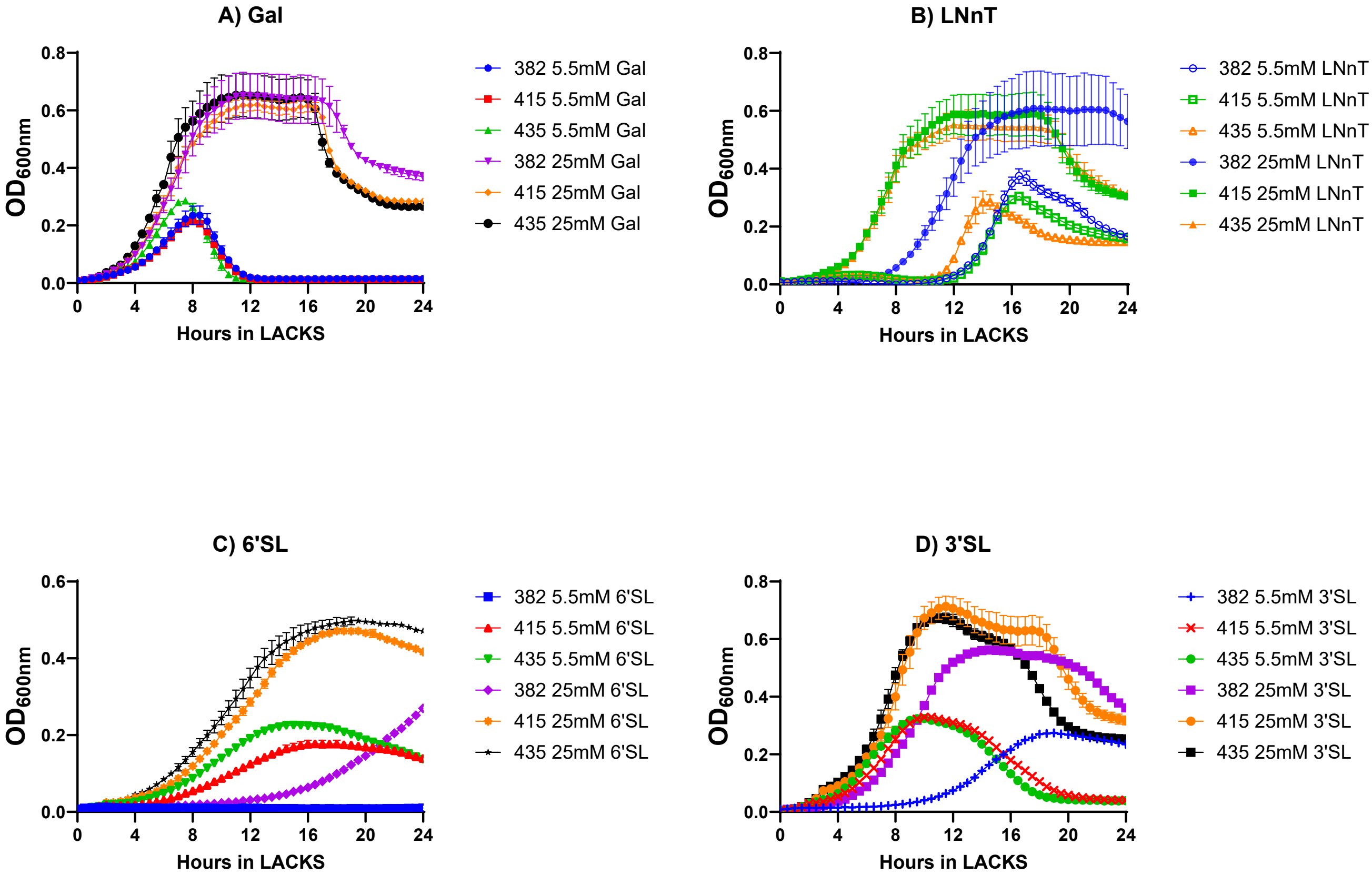

Figure S12: Pneumococcal growth curves comparing the strains, FP382 (Δ*lacE-2*), FP415 (Δ*celB)* *and FP435* (Δ*celD)* in Lacks medium. The used carbon sources were **A)** Galactose (Gal), **B)** Lacto-N-neotetraose (LNnT) **C)** 6'-Sialyllactose (6'SL) and **D)** 3'-Sialyllactose (3'SL), The means of three representative technical replicas from a single biological replicate for two different concentrations are blotted ± SEM.

Supplementary figure 13

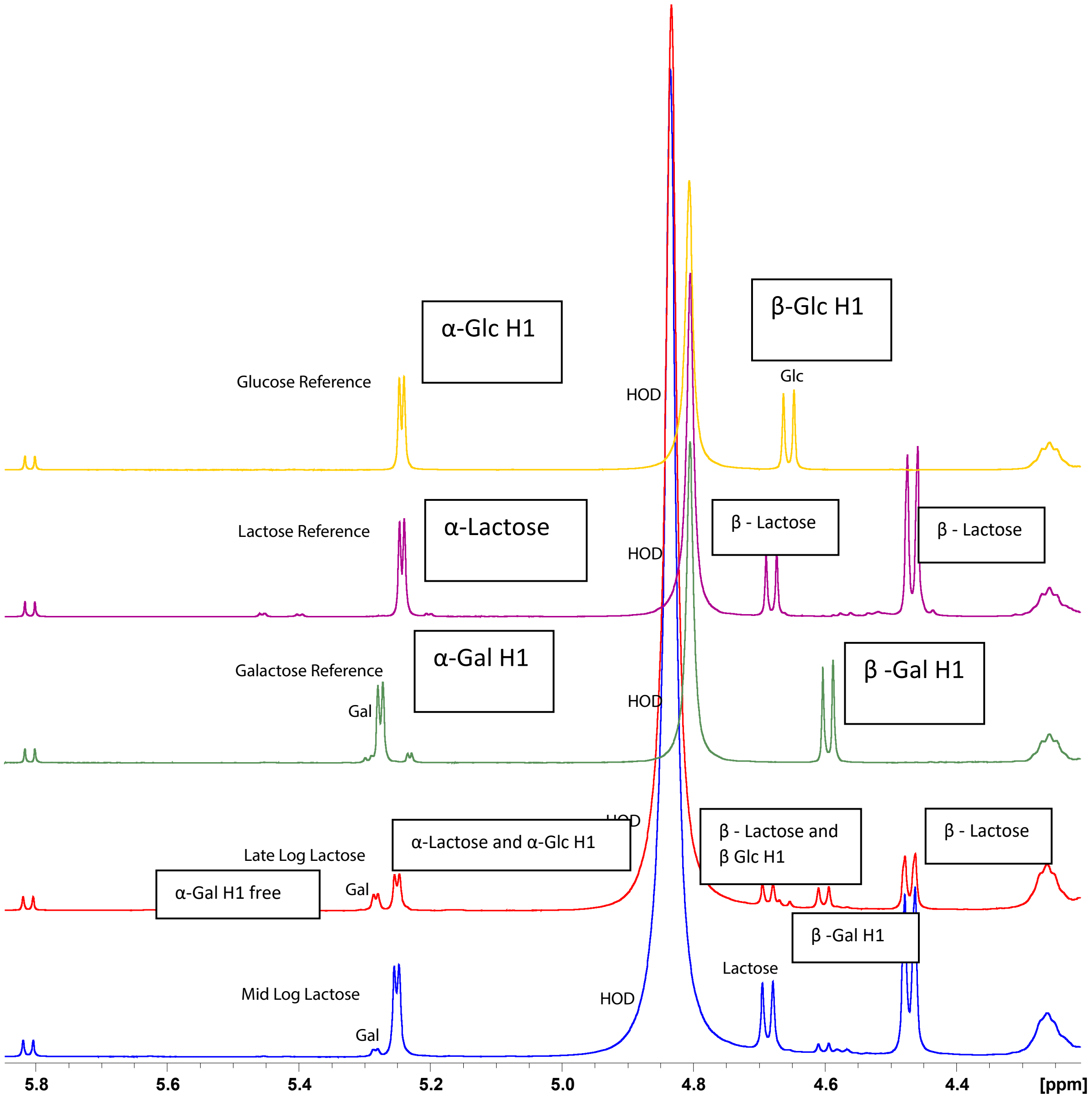

Figure S13: Excerpt of ^{1}H-NMR spectrum of pneumococcal strain grown CDM-Lactose After culture of *S. pneumoniae*, extracellular metabolite concentrations were received by ^{1}H-NMR. With increasing OD, Lactose metabolism leads to increased and decreased levels of galactose and Lactose, respectively

Supplementary figure 14

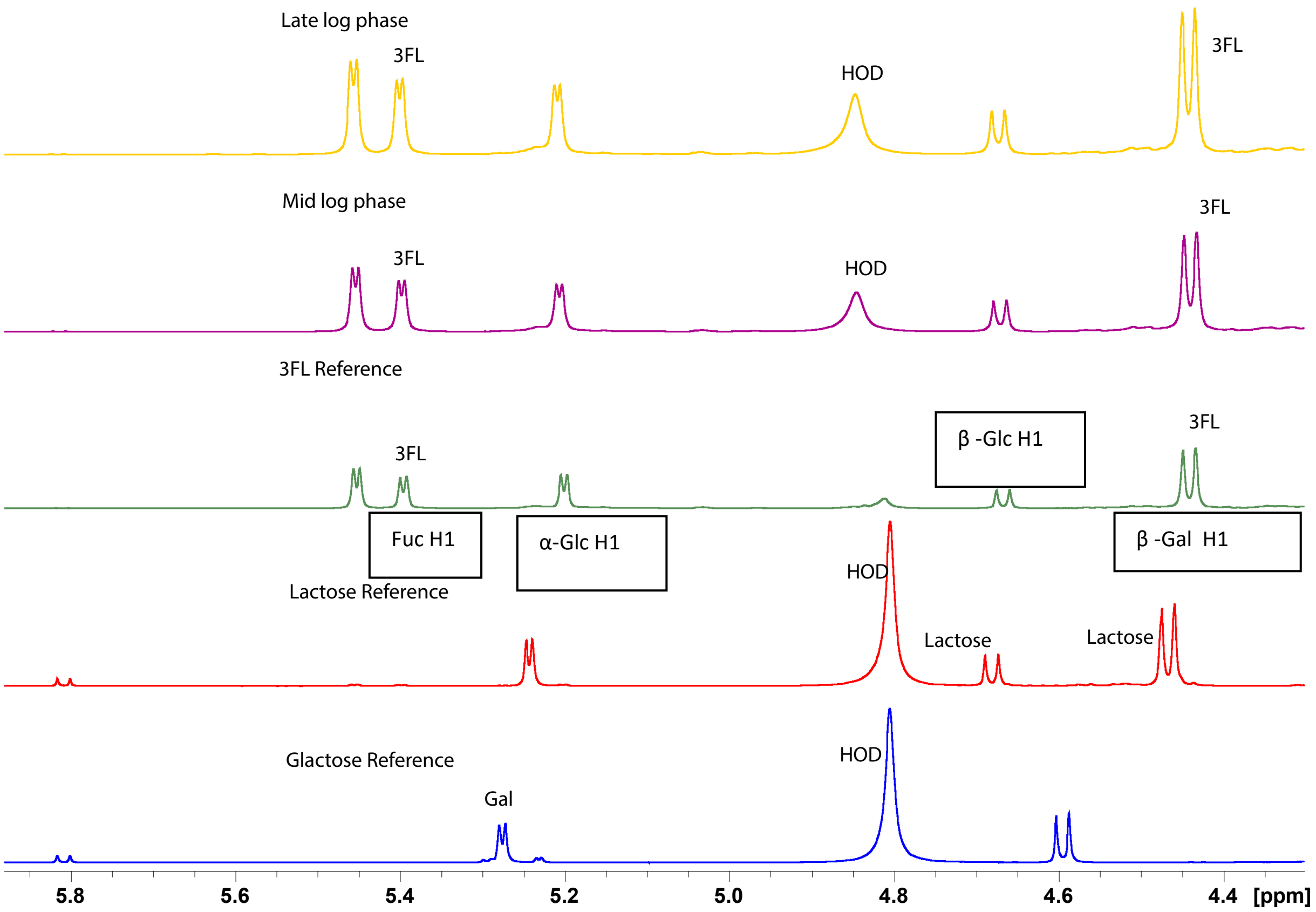

Figure S14: Excerpt of ^{1}H-NMR spectrum of pneumococcal strain grown CDM-3-FL After culture of *S. pneumoniae*, extracellular metabolite concentrations were received by ^{1}H-NMR. With increasing OD, no decreased levels of 3-FL could be observed.
