# Supplementary material for: Metabolization of human milk oligosaccharides by Streptococcus pneumoniae
Source: Nat Commun. 2026 Sep 30;17:11242. doi: 10.1038/s41467-026-77970-7 (PMC13720028; doi:10.1038/s41467-026-77970-7)
Supplement: Supplementary file 4 [file 41467_2026_77970_MOESM2_ESM.pdf]

Description of Additional Supplementary Files:

Supplementary Data 1: Purified human milk oligosaccharides provided by DMS-Firmenich (Denmark). Indicated are the purity values of the tested HMOs.

Supplementary Data 2: Annotations of pathways of selected genes from D39V.

Supplementary Data 3: Differential gene expression values for the strain D39 grown in either Lac or 6-SL (up to OD 0.4). Differential enrichment was tested using DESeq2 (negative binomial generalized linear model with a Wald test) with two-tailed tests and P-values adjusted for false discovery rate.

Supplementary Data 4: Differential gene expression values for the strain D39 grown in either Lac or LNnT (up to OD 0.4). Differential enrichment was tested using DESeq2 (negative binomial generalized linear model with a Wald test) with two-tailed tests and P-values adjusted for false discovery rate.

Supplementary Data 5: Differential gene expression values for the strain D39 grown in either Lac or 6-SL (up to OD 0.12). Differential enrichment was tested using DESeq2 (negative binomial generalized linear model with a Wald test) with two-tailed tests and P-values adjusted for false discovery rate

Supplementary Data 6: Differential gene expression values for the strain D39 grown in either Lac or LNnT (up to OD 0.12). Differential enrichment was tested using DESeq2 (negative binomial generalized linear model with a Wald test) with two-tailed tests and P-values adjusted for false discovery rate

Supplementary Data 7: Fold chain values from CRISPRi sequencing for the strain D39 for all conditions.

Supplementary Data 8: Fold chain values from CRISPRi sequencing for the strain R6 for all conditions
